# Designing a Surveillance Sensor Network with Information Clearinghouse for Advanced Air Mobility

**DOI:** 10.3390/s24030803

**Published:** 2024-01-25

**Authors:** Esrat Farhana Dulia, Syed A. M. Shihab

**Affiliations:** College of Aeronautics and Engineering, Kent State University, Kent, OH 44242, USA; sshihab@kent.edu

**Keywords:** advanced air mobility, uncrewed aircraft system, sensor placement model, optimization, surveillance information clearinghouse, cost–benefit analysis

## Abstract

To ensure safe, secure, and efficient advanced air mobility (AAM) operations, an AAM surveillance network is needed to detect and track AAM traffic. Additionally, a cloud-based surveillance data collection, monitoring, and distribution center is needed, where AAM operators and service suppliers, law enforcement agencies, correctional facilities, and municipalities can subscribe to receiving relevant AAM traffic data to plan and monitor AAM operations. In this work, we developed an optimization model to design a surveillance sensor network for AAM that minimizes the total sensor cost while providing full coverage in the desired region of operation, considering terrain types of that region, terrain-based sensor detection probabilities, and meeting the minimum detection probability requirement. Moreover, we present a framework for the low altitude surveillance information clearinghouse (LASIC), connected to the optimized AAM surveillance network for receiving live surveillance feed. Additionally, we conducted a cost–benefit analysis of the AAM surveillance network and LASIC to justify an investment in it. We examine six potential types of AAM sensors and homogeneous and heterogeneous network types. Our analysis reveals the sensor types that are the most profitable options for detecting cooperative and non-cooperative aircraft. According to the findings, heterogeneous networks are more cost-effective than homogeneous sensor networks. Based on the sensitivity analysis, changes in parameters such as subscription fees, the number of subscribers, sensor detection probabilities, and the minimum required detection probability significantly impact the surveillance network design and cost–benefit analysis.

## 1. Introduction

### 1.1. Advanced Air Mobility

AAM is envisioned to allow emerging short-haul aircraft, such as small uncrewed aircraft systems (sUAS) and electric vertical takeoff and landing aircraft (eVTOL), to operate in the lower altitudes of national airspace for passenger and cargo transportation and other use cases in the coming years. AAM is anticipated to offer a number of benefits to society and the environment over traditional ground transportation systems, including a considerable reduction in travel and delivery times, increased operational safety, and a reduced negative impact on the environment [1,2]. Federal agencies, such as NASA and FAA, and industry and academia have been focusing their research on AAM aircraft design [3], concepts of operation [4], air traffic management [5], trajectory planning [6], deconfliction [7], market studies [8,9], network planning [10,11,12], and operations planning [13,14]. A more comprehensive review of past and recent AAM research can be found in [15,16].

### 1.2. Motivation and Contributions

#### 1.2.1. Surveillance Sensor Network Design for Advanced Air Mobility

**Figure 1:**
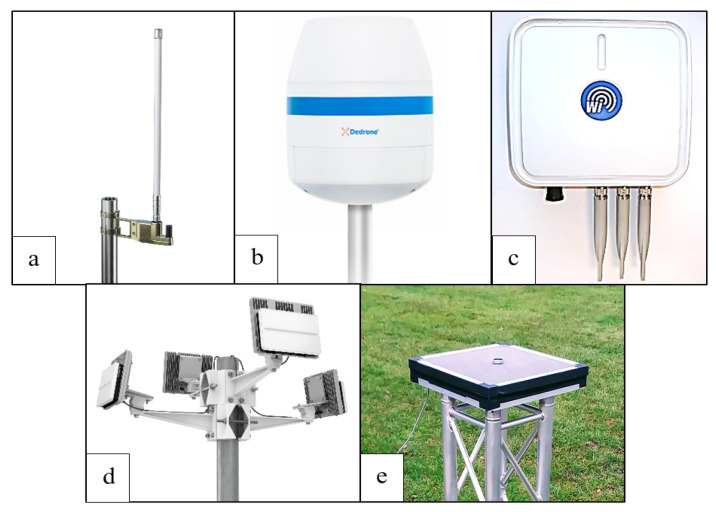
Sensors of different types: (**a**) ADS–B receiver [18], (**b**) radio frequency sensor [19], (**c**) remote ID receiver [20], (**d**) radar [21], and (**e**) acoustic sensor [22].

Motivation: While AAM research has been advancing on many fronts, one area of research that is critical to enabling AAM but has not received much attention is surveillance sensor network design for AAM. New surveillance sensor networks are specifically needed for AAM to detect and track AAM traffic to ensure efficient, safe, and secured AAM operations. Much of the existing surveillance infrastructure for conventional aviation is not adequate for AAM for mainly two reasons. Firstly, AAM is envisioned to involve operations of aircraft within new urban and suburban surveillance areas, where no sensors currently exist for aircraft surveillance. Secondly, traditional aviation sensors, which may already exist in anticipated surveillance areas for AAM, will not be adequate because they have not been designed specifically to detect small aircraft, such as sUAS and eVTOLs, or accurately identify multiple aircraft flying near each other at lower altitudes in inclement weather conditions [17], as would be the case for future high-density AAM operations. Hence, AAM-specific sensors with specialized features and capabilities are needed that enable real-time detection and tracking of AAM aircraft in various weather conditions, simultaneous detection of multiple aircraft, and accurate identification and classification of AAM aircraft. Keeping these requirements in mind, several different types of surveillance sensors for AAM—including, radar, radio frequency sensors, Automatic Dependent Surveillance–Broadcast (ADS–B) sensors, remote ID sensors, optical sensors, and acoustic sensors—have been developed by various sensor manufacturers, such as Echodyne, Dedrone, and AVIONIX. Examples of such sensors are pictured in Figure 1. These sensors use either electromagnetic or sound waves to determine the distance, angle, and radial velocity of aircraft relative to their installation sites to detect and track aircraft. Such AAM sensors need to be set up to form a surveillance network in future AAM surveillance areas. While other research has focused on designing surveillance networks for both aviation and non-aviation applications, the specific context of AAM remains largely unexplored.

Contributions: To bridge this gap, our study is the first, to the best of our knowledge, in solving the AAM surveillance sensor placement problem. In this study, we propose a Surveillance for AAM Network Design (SAND) optimization model for identifying the optimal locations for placing the sensors to build the AAM surveillance network such that (1) full coverage is provided in the desired region of operation; (2) the minimum detection probability requirement is satisfied; and (3) the total sensor cost is minimized. Our study collectively considers several features that stand out, even in comparison to relevant articles that have developed sensor location optimization models across other fields. These features include incorporating multi-type sensors with varying radii and other specifications for a heterogeneous sensor network, encompassing both the probability of detection and misdetection of sensors and considering different terrain types of a given area along with the detection probability of sensors based on terrain types. Our model can perform in various cities of Ohio, handling both large- and small-scale problems, adapting to irregular shapes of the surveillance area, and excluding infeasible blocks where sensors cannot be placed. The inclusion of critical constraints in the field of AAM, such as a minimum required detection probability and full coverage, is considered in our study. Another notable feature of our model is its ability to ensure global optimal solutions at a given minimum required detection probability value and a set of detection probabilities of sensors based on the terrain types of a city. The collective consideration of these features, which has not been done before, sets our study apart from other relevant studies.

#### 1.2.2. Low Altitude Surveillance Information Clearinghouse

Motivation: The demand for AAM is expected to grow rapidly in the coming years due to several factors, such as urbanization, population growth, and the ever-increasing need for more efficient and sustainable transportation solutions [23]. Therefore, significant amounts of AAM traffic surveillance data will be generated by the AAM surveillance network, which would require efficient storage and processing solutions to ensure that the data are easily accessible and available for real-time and offline analysis by relevant AAM stakeholders, such as AAM operators, airspace service providers, and law enforcement agencies. A digital LASIC can act as a central repository for this traffic data, allowing for data accessibility and sharing among various entities for flight planning, aircraft routing, air traffic control, counter uncrewed aircraft systems (UAS) operations planning, and better coordination among low altitude airspace users. Some of the functions LASIC can enable for its users include access to live surveillance feeds, real-time coverage maps, and archival data; data analytics and visualization; tactical deconfliction; and querying current and historical UAS positions by UAS ID and by location [24].

For implementing and hosting LASIC, a cloud server is considered to be more suitable than a local server, as cloud computing can provide a scalable, flexible, and cost-effective platform for ingesting, processing, storing, analyzing, and sharing large amounts of transportation data [25] generated by AAM traffic. Cloud computing can improve the performance and efficiency of transportation systems such as LASIC by relocating the hardware and software components to the cloud network [26], which would allow LASIC to access the computing resources and data storage capabilities of the cloud network. This can potentially reduce the need for the expensive hardware and infrastructure associated with local servers on-site while also providing greater flexibility and scalability for LASIC operations.

**Figure 2:**
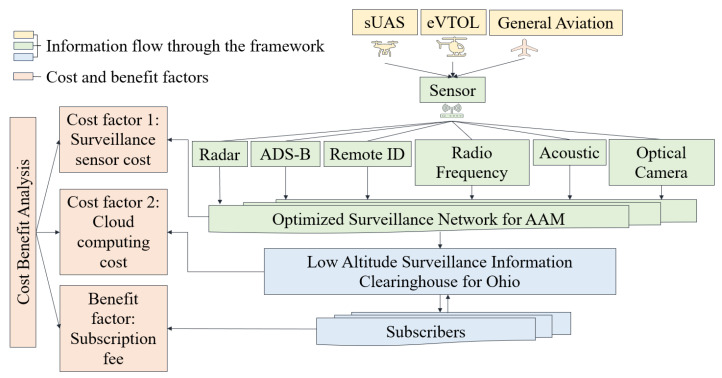
Overview of LASIC framework and associated cost and benefit factors.

Contributions: In this study, the optimized AAM surveillance network is connected to LASIC, efficiently handling AAM surveillance data generated through sensor tracking and detection of AAM traffic. This integration is considered based on AAM traffic projections, surveillance data types, interface standards, data sizes, ping rates, and cloud components. An overview of the optimized AAM surveillance network and LASIC framework and its associated cost and benefit factors are illustrated in Figure 2. Based on a survey of the present AAM sensor market, we selected six different sensor types: a radar, radio frequency sensor, ADS–B, remote ID, optical camera, and acoustic sensor. The surveillance and telemetry data associated with sUAS, eVTOL, and general aviation traffic—such as position, velocity, flight intent, and remote identification—can be captured and generated by the optimized surveillance network, allowing the aircraft movement in the airspace to be tracked. This surveillance data can then be ingested into LASIC, which will provide the subscribers of LASIC with information about scheduled and real-time AAM operations and relevant airspace activities so that they may plan for their flight operations accordingly. The subscribers of LASIC will potentially include AAM operators engaged in different AAM use cases such as passenger and cargo transportation, bridge inspections, medical delivery, etc., as well as airspace service providers, law enforcement agencies, correctional facilities, and municipalities.

As for any other major transportation infrastructure project, to justify an investment in the AAM surveillance network and LASIC, a rigorous cost–benefit analysis is needed [27,28,29]. Such an analysis is crucial to identify, quantify, and evaluate the costs and benefits associated with the surveillance network and LASIC. We conducted this cost–benefit analysis for the State of Ohio by analyzing the associated cost and benefit factors of LASIC. The analysis period was considered to be the next 10 years, from 2024–2033. The two major cost factors of LASIC considered are as follows: (1) the surveillance sensor cost, the cost to purchase the sensors needed in the AAM surveillance areas in Ohio, which we estimate based on the results generated from the SAND model, and (2) the cloud computing cost to store and process the surveillance data in LASIC. The monthly subscription fee that a subscriber will pay to obtain access to the LASIC features is considered the main benefit factor in this analysis. The cost–benefit analysis can be used to estimate the break-even point (BEP) for the different sensor types and the time to reach break-even in terms of the net present value (NPV) of the return generated in the AAM surveillance areas.

#### 1.2.3. Summary of Contributions

This paper addresses the critical need for a surveillance network design in the rapidly emerging landscape of AAM, offering insights into sensor selection, network optimization, data management, and economic feasibility. Our key contributions are as follows:

(a) We developed the SAND model, which can determine the optimal locations for sensor deployment to design a comprehensive AAM surveillance network that minimizes the total sensor cost. The SAND model can provide full coverage in the desired AAM surveillance areas and considers terrain types within those regions, as well as terrain-based sensor detection probabilities and minimum detection probability requirements. We considered the State of Ohio as our case study and applied the SAND model to design an AAM surveillance network there.
(b) We considered several sensor types, such as radar, radio frequency, ADS–B, remote ID, optical, and acoustic sensors, to design two types of AAM surveillance sensor networks: homogeneous and heterogeneous. Our analysis of homogeneous sensor placement indicates that ADS–B and remote identification sensor types are the most profitable options for detecting cooperative aircraft, whereas the radio frequency sensor type is the most profitable option for tracking both cooperative and non-cooperative aircraft. According to the findings, implementing a heterogeneous sensor network composed of various sensor types is more cost-effective in reducing the overall sensor cost compared to a homogeneous sensor network that employs only one type of sensor.
(c) We present a cloud-hosted LASIC framework, which allows for the managing and sharing of AAM surveillance traffic data. We computed the cost of operating the framework while considering the AAM traffic projections and relevant surveillance data generated in the AAM surveillance areas in Ohio, as well as the surveillance data types, interface standards, data sizes, cloud components, and cloud pricing policies.
(d) We conducted a rigorous cost–benefit analysis of the proposed AAM surveillance network and LASIC implementation for the State of Ohio to determine the break-even points for different sensor types. We considered the uncertainty associated with AAM demand to determine the possible range of costs, revenue, and NPV for the AAM surveillance network and LASIC.
(e) We performed a sensitivity analysis on the key parameters of our study, including the subscription fees, number of initial subscribers, terrain-based sensor detection probabilities, and minimum required detection probability. The insights demonstrate that changes in these parameters significantly impact the number of sensors required, total sensor cost, and NPVs of the results generated from the study. Our study provides policymakers with valuable insights to make informed decisions regarding investment in an AAM surveillance network and LASIC.

### 1.3. Outline of the Paper

The remainder of this article is structured as follows. The relevant literature is analyzed in Section 2. In Section 3, the SAND model is presented, and the potential cost and benefit factors of LASIC are discussed. After that, the results are presented and analyzed in Section 4. The paper is finally concluded in Section 5 with the summary of insights gained from the analysis and potential extensions of this study.

## 2. Literature Review

A review of the research on AAM surveillance and the general surveillance network design problem is presented in this section. For a more broader overview of the AAM research, interested readers are referred to [15,16], where the authors collectively discussed prior AAM research and unresolved AAM challenges related to aircraft specifications, regulations, certification, policy, demand modeling, traffic management, ground infrastructure, operational strategies, market structures, integration with existing transportation systems, and public acceptance.

### 2.1. AAM Surveillance

A number of recent studies in the literature have concentrated on surveillance technologies, frameworks, and simulations aimed at tracking and monitoring AAM aircraft. Notably, NASA’s recent work [30] has emphasized the necessity for surveillance of AAM aircraft, underlining the difficulty of modifying present air traffic control and management systems to accommodate the increased number of AAM aircraft in the lower airspace. In response, they have introduced a ground-based vision tracker that employs a vision tracking method with fixed cameras to monitor airborne objects, effectively sidestepping issues related to electromagnetic interference. Additionally, ref. [31] developed a simulation system to model and assess AAM flight operations in densely populated urban areas using both air- and ground-based sensors such as radar, LiDAR, and vision-based sensors. The purpose of this paper was to present the architecture and simulation setup for evaluating airborne autonomy technologies for urban AAM operations. In [32], the authors mentioned remote identification as an emerging technology that allows ground observers to identify drones within airspace. Their objective was to provide a comprehensive overview and tutorial on the current status of regulatory, standardization, design, implementation, and testing efforts in the field of remote identification technology. In another study [33], the authors developed a surveillance framework to address the growing security threats facing critical infrastructures such as airports, military bases, city centers, and other restricted zones. This framework utilizes radio frequency (RF) sensors to efficiently detect, classify, and identify drones operating within no-drone zones. However, comprehensive research that explores various types of AAM surveillance networks, taking into account diverse requirements, such as tracking different types of AAM aircraft and the use of different types of sensors to build an AAM surveillance network, has not yet received sufficient attention. While research has been conducted on state-of-the-art sensor types related to ground-based detect-and-avoid systems for UASs, as presented in [34], these sensor types have not been thoroughly examined to develop different types of AAM surveillance networks. In this study, we investigate six potential sensor types suitable for AAM surveillance and assess the different types of surveillance sensor networks: homogeneous sensor networks consisting of a single sensor type and heterogeneous sensor networks consisting of various sensor types. Additionally, we conduct a cost–benefit analysis of the AAM surveillance network to provide justification for investing in AAM surveillance infrastructure.

### 2.2. Location Selection Problems and Surveillance Network Design

The motivating application of the SAND model is the AAM surveillance network design, which involves solving a sensor location selection problem. In general, the location selection problem is concerned with determining the best locations for new facilities and services with respect to performance metrics such as cost, revenue, profit, travel time, distance, customer satisfaction, etc. Such problems arise in various fields, such as remote sensing, geography, economics, and operations research [35]. Researchers and practitioners have developed various techniques to address the location selection problem, which primarily includes multi-criteria decision-making (MCDM), machine learning (ML), heuristics, metaheuristics, and mathematical optimization.

*Multi-Criteria Decision-Making*: MCDM is a well-known approach that is used for tackling location selection problems. As the name suggests, MCDM determines the optimal location for various types of facilities—such as a new manufacturing plant, a retail store, a hospital, a distribution center, a transportation hub, or a renewable energy facility—based on multiple criteria or objectives. In the context of location selection problems, the criteria can include factors such as proximity to suppliers or customers, transportation costs, availability of labor, and many other factors that can affect the desirability of a location. The use of MCDM in location selection problems has been extensively studied in the literature. Three such representative papers are discussed next. The locations of manufacturing facilities were determined using MCDM in [36], taking into account criteria such as access to raw materials, labor force, and transportation infrastructure. This study considered several criteria, such as economic, environmental, and societal factors, in their facility location selection problem for sustainable development in manufacturing firms. An analytical hierarchy process (AHP) was also used here to evaluate the weights of these criteria, and a technique for order preference by similarity to ideal solution (TOPSIS) was used to rank the alternative potential locations. MCDM was also used to determine the best locations for hospitals and clinics, taking into account factors such as patient population, access to public transportation, and proximity to other healthcare providers. A location selection problem was solved in [37] using a MCDM approach to find the emergency medical service centers. In this study, AHP was used to determine the weights of criteria, including response time, demand, coverage area, and ambulance workload. Then, the different alternative locations of service centers were ranked using a technique known as ranking of alternatives through the functional mapping of criterion sub-intervals into a single interval (RAFSI). A MCDM approach based on a fuzzy approach was presented in [38] for determining the location of healthcare facilities. While the fuzzy logic approach can model complex systems using linguistic variables and fuzzy sets, it does not, however, guarantee an optimal solution. MCDM techniques, such as AHP, TOPSIS, and RAFSI, rely on human judgment to evaluate and weigh the criteria. There may be differences in opinion or interpretation among the decision-makers, which can lead to inconsistent or biased results, particularly if the criteria are not well-defined or if the weighting scheme is not properly calibrated. Hence, MCDM may not consistently yield the optimal locations. In contrast, mathematical optimization can consistently determine the optimal solution for the location selection problem by utilizing a mathematical model that adequately captures all relevant preferences and constraints.

*Machine Learning*: In recent years, ML techniques have been increasingly applied to solve location selection problems. Next, three papers are presented that applied ML techniques to solve logistic hub and sensor location selection problems. A ML-based algorithm framework was proposed to solve hub location problems in the logistics and transportation industries. This framework consisted of a deep-learning probabilistic hub-ranker that ranks the priority of nodes to be chosen as hubs [39]. To evaluate the effectiveness of this approach, the study created 11,000 small networks, each with 25 nodes, using a proposed data augmentation technique. These synthetic networks were divided into three sets: 10,000 for training models, 500 for validation during training to prevent over fitting, and 500 for model evaluation. Ref. [40] presented a ML-based method for optimal sensor placement in the flow over an airfoil equipped with a Coanda actuator. The method utilized a random forest algorithm to construct ML models that predicted a response function based on input data from 96 sensors measuring pressure and skin friction coefficients. The optimal sensor positions were determined by identifying the most important input variables in the ML model. A limitation of the method was its reliance on many sensors during the training phase, which made it challenging to implement experimentally. An ML algorithm was proposed and implemented in [41] to select optimal sensor locations in controlled environment agriculture, where the macro-climate affects the micro-climate, making it challenging to predict the ideal conditions. The algorithm used temperature and humidity data from 56 different locations that were collected over a year, processed to remove outliers, and transformed to other air properties. The results showed that three to five sensors were needed, and there were similar sensor locations for different air properties. While ML has shown great promise in solving complex optimization problems, such as the location selection problem, it has some drawbacks compared to mathematical optimization methods. One potential drawback is that ML models are typically designed for specific problem settings and may not be easily adaptable to other problem settings or variations, which can be a disadvantage when dealing with a new, evolving field like AAM. Though retraining ML models is possible for adapting them to new problem settings, this process can be time-consuming and computationally expensive, which may limit the practical usefulness of these approaches in dynamic and rapidly evolving fields. Another challenge is that ML models require large amounts of data to be trained effectively, and the scarcity of AAM-related training data can hinder their applicability. In contrast, mathematical optimization methods are highly flexible and can be customized to suit a wide range of problem settings by adjusting the objective function, decision variables, and constraints to model the new problem. Moreover, unlike ML models, mathematical optimization methods provide guarantees for generating optimal solutions. Given the limited availability of AAM-related data and the need for optimal solutions, mathematical optimization methods appear to be a more suitable option for addressing location selection problems in emerging transportation sectors such as AAM.

*Heuristics and Metaheuristics Algorithms*: Heuristic and metaheuristic algorithms are optimization algorithms that have also been used to address location selection problems due to their ability to efficiently handle complex and large-scale problems. A proposed theory was described in [42] for optimizing the placement and number of sensors in a sensor network using a greedy heuristic. The sensor field was represented as a grid of points, and the optimization framework addressed coverage optimization under constraints of imprecise detection and terrain properties. The article explained how obstacles in the terrain were modeled in the framework, and the sensor placement algorithm used a greedy heuristic to determine the best placement of one sensor at a time. The algorithm was iterative and terminated either when a preset upper limit on the number of sensors was reached or when sufficient coverage of the grid points was achieved. To solve the problem of locating fire stations and allocating resources to different stations based on dynamic traffic conditions, two metaheuristic algorithms—particle swarm optimization and artificial bee colony—were used in [43]. In another study, ref. [44] used a genetic algorithm to optimize the placement of security cameras, providing maximum coverage of user-defined priority areas and minimizing the probability of occlusion due to moving objects by covering each priority area with multiple cameras. Ref. [45] proposed a solution for determining the optimal placement of ADS–B receivers on the ground. In [45], a genetic algorithm was utilized to determine the optimal placement of ADS–B receivers on the ground in the vicinity of Frankfurt Airport in Germany. The authors first identified the required number of sensors to ensure adequate coverage of the small geographical area. The algorithm was designed to search for the best local minimums or near-optimal solutions. Then, the authors quantified the deviation of the sensor configuration generated by the algorithm from the optimal solution. Ref. [46] also implemented a metaheuristic algorithm for solving optimal sensor placement problems using an annealing machine. One major drawback of such heuristic and metaheuristic algorithms compared to mathematical optimization is that they do not guarantee to find the global optimal solution. They involve stochastic search processes, which means that they may converge to a sub-optimal solution depending on the starting point and the algorithm parameters.

*Mathematical Optimization*: Mathematical optimization is a widely used approach to solving location selection problems and aims to identify the best solution by minimizing or maximizing an objective function subject to a set of constraints. The advantage of using mathematical optimization is that it allows us to find the optimal solution that satisfies all the constraints with high precision and efficiency. Ref. [47] presented a decision-making process to select the location for public truck parking lots in urban areas using mixed-integer programming. The process included candidate location selection by spatial analysis and optimal location determination using the competitive p-median algorithm. A constrained multi-objective optimization problem with mixed-integer programming was developed in [48] to simultaneously determine the placement of wireless sensors and sinks that minimize energy consumption and maximize information effectiveness for structural health monitoring (SHM). Ref. [49] provided an overview of the state-of-the-art in the area of optimization of sensor placement for SHM applications. The optimal sensor placement problem addressed in [50] aimed to select appropriate types and locations of sensors that could cover high-value terrain areas while minimizing a cost function. The probability of detection was assumed to depend on terrain conditions and obstructions. Two strategies were used for optimal sensor placement: the initial strategy utilized a heuristic and fast approach that involved placing sensors one-by-one in the location where they were most needed, while the second strategy was a binary linear programming solution that determined the global optimum of the total cost of sensors, without allowing for the sequential placement of sensors. After evaluating the strengths and limitations of the various methods discussed above, mathematical optimization was determined to be the most suitable method for addressing the surveillance network design problem of AAM. This preference is primarily due to its capability to guarantee global optimal solutions, even in the dynamic nature of the AAM market. The AAM data can be easily adjusted in the model’s parameter setting to update the AAM surveillance network as the field evolves.

### 2.3. Our Contributions

The location selection problem is addressed in various fields, but to our knowledge, we were the first to solve the AAM surveillance sensor placement problem. Given that the optimization approach is identified as the most appropriate method to address the gap, we propose the SAND model, which can generate solutions for building an optimized AAM surveillance network. Our study stands out by collectively incorporating 12 features, as outlined in Table 1, compared to other relevant studies where authors have used optimization to address the location selection problem, ensuring sensor coverage over specific areas. The first feature involves considering multi-type sensors to utilize the characteristics of various sensors in developing a heterogeneous sensor network, providing coverage at a lower cost. Among the relevant studies, only [50] considered this feature, but that study focused on three sensor types, whereas our study incorporates twice as many, including six sensor types. The second feature of our study involves considering the probability of the misdetection of sensors, a crucial parameter that most of the studies did not take into account. This feature influences the optimal number of sensors required to provide sensor coverage over a given surveillance area. Unlike most studies, our study considers different terrain types, such as hills, towns, and water, within the surveillance area, along with the detection probability of a sensor type based on these terrain types.

To ensure the versatility of our SAND model across various surveillance areas, we incorporated the fourth feature in our study, which involves implementing the model in six different cities with varying sizes and combinations of terrains. Another study, ref. [51], considered this feature for two cities, which is three times fewer than ours. In contrast, other studies tested their models in only one city, specific areas of a city, or hypothetical areas without considering real-world locations. This raises concerns about the flexibility of their models to adapt to the diverse characteristics of different cities. The fifth feature of our study is the consideration of a higher number of blocks dividing the surveillance area compared to most studies. Considering this higher number of blocks allows us to increase the resolution of the surveillance area—although it increases the complexity and runtime of the model. To avoid this complexity, models developed in most studies can handle only a lower number of blocks, potentially failing to generate solutions within a finite time for larger-scale problems. Our model, however, can generate solutions for both higher- and lower-scale problems. We verified this feature by testing our model in different sizes of cities, creating a higher number of blocks for a larger city and a lower number of blocks for a smaller city. The sixth feature of our study is the adaptability of an irregular shape of a surveillance area. The studies presented in Table 1 considered rectangular meshes to define the surveillance area but did not ensure the exclusion of blocks that were not within the surveillance area. However, the surveillance area can have irregular shapes. Our SAND model can closely resemble the actual shape of the surveillance area, thereby improving the solutions generated compared to other studies. Unlike some studies, our study considers excluding infeasible blocks, for example, water blocks, where the sensor cannot be placed, from candidate sensor locations. This is the seventh feature of our study.

Similar to [42,50], we also incorporated a minimum required detection probability constraint in our study, which is the eighth feature of our study. In the field of AAM, compared to any other field, a minimum required detection probability value, for example, 95–99%, is considered to ensure a higher level of detection and tracking accuracy. This consideration increases the complexity of the model significantly, but our SAND model satisfies this constraint as well. The ninth feature of our study incorporates a full coverage constraint to ensure sensor coverage over the entire surveillance area at a given minimum required detection probability value. Refs. [42,52] also considered achieving coverage by assuming that all block points are covered, but this approach still has the chance of gaps between two sensors. While the model in [42,52] provided full coverage, it lacked efficiency in terms of computational time, resulting in unnecessary overlapping initially. In contrast, our SAND model incorporates a dedicated constraint to provide an efficient approach, reducing the algorithm runtime. The tenth feature is that the SAND model guarantees global optimal solutions at a given minimum required detection probability value and a set of detection probabilities of sensors based on the terrain types of a city. Most of the studies apply greedy heuristics [42], simulated annealing algorithms [53], genetic algorithms [52], sparrow search algorithms [54], or both the simulated annealing algorithm and genetic algorithm [51] to solve the sensor placement problem. While these algorithms have their advantages, one of their limitations is the inability to guarantee global optimal solutions, as discussed earlier. Although [50] guarantees global optimal solutions, that study did not consider the fourth, sixth, and ninth features and included fewer blocks than our study. In contrast, our SAND model ensures global optimal solutions while collectively considering all the features discussed above. The eleventh feature involves connecting the AAM surveillance network to LASIC for receiving, storing, and processing AAM surveillance data, enabling AAM service providers to operate their operations accordingly. We take into account AAM traffic projections, surveillance data generated from the AAM traffic detected and tracked by the sensors, surveillance data types, interface standards, data sizes, ping rate, and cloud components in considering this connection. The last feature involves conducting a cost–benefit analysis to justify the investment in the AAM surveillance network and LASIC infrastructure. The unique combination of these features, not previously explored collectively, distinguishes our study from other relevant research.

**Table 1:** Comparison of our study with other relevant studies.

	Features	[42]	[50]	[53]	[52]	[54]	[55]	[51]	Our Study
1	Multi-type sensors	X	✓	X	X	X	X	X	✓
2	Probability of misdetection of sensors	✓	✓	X	X	X	X	X	✓
3	Detection probability of sensors based on terrain types	X	✓	X	X	X	X	✓	✓
4	Multi-city	X	X	X	X	X	X	✓	✓
5	Maximum number of blocks	20 × 20	81 × 81	30 × 30	90 × 90	50 × 100	90 × 90	120 × 200	130 × 126
6	Adaptability to irregular shape of surveillance area	X	X	X	X	X	X	X	✓
7	Exclusion of infeasible blocks from candidate sensor locations	X	✓	X	X	✓	X	✓	✓
8	Minimum required detection probability constraint	✓	✓	X	X	X	X	X	✓
9	Full coverage constraint	X	X	X	X	X	X	X	✓
10	Guarantee of global optimum solutions	X	✓	X	X	X	X	X	✓
11	Connection to LASIC	X	X	X	X	X	X	X	✓
12	Cost–benefit analysis of AAM surveillance network and LASIC infrastructure	X	X	X	X	X	X	X	✓

## 3. Methodology

**Figure 3:**
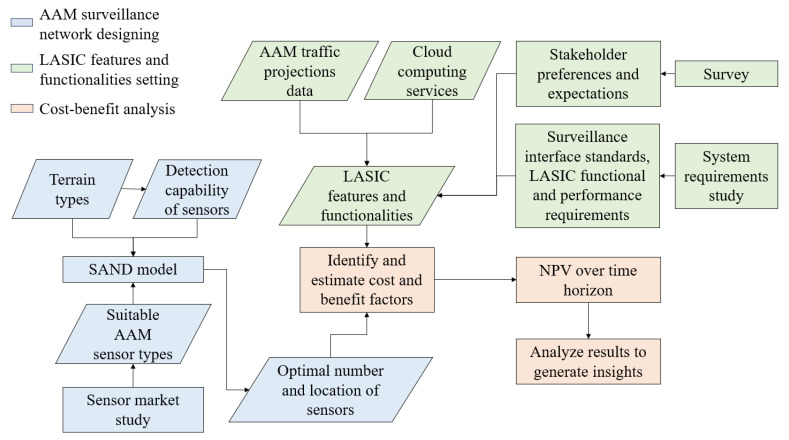
A flow chart illustrating the steps associated with AAM surveillance network designing and cost–benefit analysis of AAM surveillance network and LASIC.

This section aims to develop the SAND model for designing an AAM surveillance network and also considers a framework for LASIC to receive surveillance data from the optimized AAM surveillance network. We then conduct a cost–benefit analysis to justify the investment in the AAM surveillance network–LASIC infrastructure. An outline of our methodology is presented in Figure 3, which is divided into three parts: the first part involves designing the AAM surveillance network, the second part involves setting LASIC features and functionalities, and the third part involves conducting a cost–benefit analysis of the AAM surveillance network and LASIC. The blue boxes represent the inputs of the SAND model, including AAM sensor types, terrain types, and sensor detection probabilities, as well as its outputs, such as the optimal number and location of sensors, which are further discussed in Section 3.1. The green boxes correspond to the inputs LASIC feature and functionalities, including AAM traffic projections data, cloud-computing services, and insights from the survey and system requirements study that are elaborated upon in Section 3.2 and Section 3.3.2. The orange boxes represent the steps associated with the cost–benefit analysis of the AAM surveillance network and LASIC. The results of the first and second parts are utilized to identify and estimate the cost and benefit factors, which are discussed in detail in Section 3.3 and Section 3.3.3, respectively. The output of the cost–benefit analysis is the NPV over the analysis period, which is explained in Section 3.3.4.

### 3.1. Surveillance Sensor Network Design for Advanced Air Mobility

We constructed a BILP model to solve the optimal sensor placement problem for designing the surveillance network for AAM. The objective of this study is to design a network of surveillance sensors to track AAM aircraft flying at a lower altitude over a city with a minimum sensor cost while satisfying two constraints: (1) sensor(s) at a location must provide a minimum required detection probability, and (2) the area across a city must be fully covered by the network. Given these restrictions, the goal is to determine the optimal locations for sensor placement and the number of sensors needed to be placed within a city to detect and track the aircraft, where the objective function focuses on minimizing the total sensor cost.

This section highlights the crucial terms that must be taken into consideration while formulating the SAND model, such as the various types of AAM surveillance sensors, the detection probability of a sensor, and the impact of terrain types on the sensor detection probabilities. Subsequently, we present the formulation of the SAND model by modeling the relevant surveillance area where the model is implemented. A summary of the notations used in designing the SAND model is presented in Table 2.

#### 3.1.1. Surveillance Sensor Types

After studying the existing sensor market, six types of sensors are deemed to be suitable for AAM traffic surveillance. A brief overview of these surveillance sensor types is provided in this section.

1. Radar: Both cooperative and non-cooperative aircraft can be detected and tracked using ground-based radars. The radar transmits the electromagnetic waves signal towards aircraft, which bounce off the aircraft and create a detailed image of its size, shape, and location. The radar cross-section (RCS) signature of each aircraft type is distinctive, which leads to varying reflection patterns of radio waves. The radar utilizes these patterns to identify the aircraft type and determine its position, velocity, and travel direction.
2. Automatic Dependent Surveillance–Broadcast: Automatic Dependent Surveillance–Broadcast (ADS–B) is a surveillance system that allows an aircraft to periodically broadcast and track its location via satellite navigation. Currently, the FAA acknowledges ADS–B as a key enabler for trajectory-based air traffic management in the future.
3. Remote ID: The ability of sUAS and eVTOL to broadcast identification and location data during flights is known as *remote identification (remote ID)*.
4. Radio Frequency: Like the radar, the RF sensor is also able to accurately detect and categorize aircraft. However, RF sensors can detect and track small drones that may not be detectable by radar, particularly at low altitudes where the radar signal may not reflect off the drone as effectively as it would off a larger aircraft. Also, RF sensors can be more effective than radar in urban or cluttered environments, where there may be many buildings, trees, and other obstacles that can reflect or absorb radar signals. RF sensors are less affected by these obstacles because their signals can penetrate walls and other structures, making them useful for monitoring drones in indoor or urban environments. The key advantages of the RF sensor system include its low cost, ease of installation, and simplicity of integration with several other sensors, including cameras and radars.
5. Acoustic: An audio pattern that is transmitted by an aircraft’s propeller can be detected by acoustic sensors and used for aircraft positioning and classification. It uses passive acoustic sensor technology with no RF emissions, whereas the solid-state sensor is an array module that includes digital microphones and digital processors.
6. Electro-Optical/Infrared Camera: An electro-optical/infrared (EO/IR) system is a type of electronic equipment that combines electro-optical and infrared sensors to offer accurate optical information of air traffic in the airspace within its coverage range at any time. EO/IR systems can be used to carry out object tracking, assess threats from a certain distance, or monitor other aircraft or ground obstructions that must be avoided.

#### 3.1.2. Detection Probability of Sensors and Terrain Types

Detection probability is a crucial performance parameter for sensors, representing the probability of a sensor detecting an object within its field of view. Terrain types of an area, such as hills, open spaces, and water bodies, can significantly affect the detection probability of a sensor by obstructing its field of view and reducing its detection range. Different terrain types can have a range of effects on the sensor detection probabilities, with some terrain types having a greater impact than others. For example, hilly terrain types can have a substantial impact on the detection probability of sensors due to their obstruction, while open spaces can provide ideal conditions for achieving higher sensor detection probability values. To obtain the sensor detection probability values for sensors, there are two primary methods. The first method involves using manufacturer-provided data based on laboratory testing and simulations. Sensor manufacturers can provide data on detection probability for different terrain types in sensor data sheets. This data can be used to estimate the sensor’s performance in various environments. The second method involves testing sensors on-site in the specific terrain type.

#### 3.1.3. SAND Model Formulation

In developing the SAND model, the processes of modeling a surveillance area and location selection are critical in designing an effective AAM surveillance network. The process of modeling the surveillance area involves identifying the surveillance area and determining feasible sensor locations while also considering that not all locations within the surveillance area may be feasible, such as those located over water bodies. This process optimizes the placement of sensors by accurately capturing the terrain types of an area and assessing their impact on sensor placement and detection probabilities. Following this, the location selection process determines the optimal number and locations of sensors within the surveillance area, satisfying the objective and constraints, as detailed in Section 3.1. This study integrates these processes to develop the SAND model, and the steps associated with the SAND model’s development are discussed next.

##### Mesh Generation and Coordinate Transformation

To start the modeling of the surveillance area, first, a rectangular mesh, denoted by *M*, is used to divide a given area that needs AAM surveillance into a set of points and small square blocks. To create such a mesh, the city is first overlaid by a rectangle defined by four GCS points on a world map, labeled *A*, *B*, *C*, and *D*. However, the use of latitude and longitude to define locations in GCS coordinates means that the distances on the Earth’s surface can result in blocks of unequal size and non-parallel mesh lines in *M*. To address this issue, it is necessary to use a PCS that maps the Earth’s surface to a 2D Cartesian plane when creating a mesh for an area. This ensures that *M* is created with equal-sized blocks and straight, parallel mesh lines, which is important in the optimal sensor placement problem for accurate measurements and simplified visualization and analysis. In Equation (1), *F* represents the transformation function of the GCS coordinates to PCS coordinates, and $\lambda_{p}$ and $\phi_{p}$ represent the longitude and latitude, respectively, of the *p*-th point in GCS. The output of the transformation is the corresponding $\left(x_{p},y_{p}\right)$ PCS coordinates.

(1) $$\left(x_{p},y_{p}\right)=F\left(\lambda_{p},\phi_{p}\right),\;p\in \left\{A,B,C,D\right\}$$

(2) $$n_{a}=\left\lceil \frac{L_{a}}{L}\right\rceil ,n_{b}=\left\lceil \frac{L_{b}}{L}\right\rceil ,\mathrm{subject}\mathrm{to}\rho \geq \frac{L}{\sqrt{2}},$$

where $n_{a}$ and $n_{b}$ specify the number of points along the x-axis and y-axis.

##### Mesh Parameters and Block Set

The parameters $n_{a}$ and $n_{b}$ are determined by the length of the area along the horizontal axis, $L_{a}$, and the length of the area along the vertical axis, $L_{b}$, respectively, as well as by the block side length, *L*. The diagonal of a square block is represented by $\sqrt{2}L$, with the center point of a block being considered as the potential location for placing a sensor. Hence, the distance from the sensor location to a corner point of the block is $L/\sqrt{2}$. If this distance is greater than the sensor coverage range denoted by ρ, the block will not be covered by the sensor. Thus, $\rho \geq \frac{L}{\sqrt{2}}$ ensures that each block is adequately covered by the sensor(s) and to avoid blind spots or gaps in the coverage of the AAM surveillance network. The resulting mesh *M* consists of $n_{a}\times n_{b}$ points, denoted by $P^{M}$, where $\left(x_{i},y_{i}\right)$ corresponds to the *i*-th point on the 2D map. Therefore, we can express the set of all points in *M* as

(3) $$P^{M}=\left\{\left(x_{1},y_{1}\right),\left(x_{2},y_{2}\right),\ldots ,\left(x_{n_{a}\times n_{b}},y_{n_{a}\times n_{b}}\right)\right\}.$$

The set of blocks $Z^{M}$ within *M* is defined by

(4) $$Z^{M}={\left[\left[P_{j\times n_{a}+k}^{M},P_{j\times n_{a}+k+1}^{M},P_{\left(j+1\right)\times n_{a}+k}^{M},P_{\left(j+1\right)\times n_{a}+k+1}^{M}\right]\right]}_{j=1,k=1}^{n_{b}-1,n_{a}-1},$$

where the number of blocks in *M* is $\left(n_{a}-1\right)\times \left(n_{b}-1\right)$. For each adjacent point pair in *M*, a block with four corner points is created using *j* and *k*, the indices of $n_{a}$ and $n_{b}$, respectively, such that $1\leq j<(n_{b}-1)$ and $1\leq k<(n_{a}-1)$. Let $Z^{M}$ be a set of all blocks in *M*, and *z* be an index used to iterate through $Z^{M}$, where *z* ranges from 1 to $\left(n_{a}-1\right)\times \left(n_{b}-1\right)$.

##### Terrains and Sensor Detection Probabilities

The probability of detecting an AAM aircraft flying over a given block by a given sensor type is determined by the terrain type of that block. Let *T* be a set of terrain types associated with each block in $Z^{M}$ within the mesh for a given area, and *S* be a set of potential sensor types. The detection probabilities for all combinations of terrain types and sensor types is represented by the matrix $\omega_{T}^{s}$. $T_{z}$ represents the terrain type of the *z*-th block in *T*, and *s* is the index for the sensor-type set *S*. The probability of detecting an AAM aircraft, denoted by $\omega_{z}^{s}$, with a sensor of type *s* on block $z\in Z^{M}$ is represented as follows:

(5) $$\omega_{z}^{s}=\omega_{T_{z}}^{s},\;\forall z\in Z^{M},\forall s\in S,$$

where block *z* has the terrain of type $T_{z}$.

##### Exclusion of Outer Blocks

As the surveillance area of interest will likely have an irregular shape, some of the outer blocks of the rectangular mesh will not belong to the area. These outer blocks are removed from the block list $Z^{M}$. The remaining set of blocks present within the area is represented as $Z=z\in Z^{M}|I\left(z\right)=1$, where I(z) is an indicator function that equals 1 if the block *z* belongs to the area, and 0 otherwise. The number of blocks that are removed from $Z^{M}$ is

(6) $$Q=\left|\left\{z\in Z^{M}|I\left(z\right)=0\right\}\right|,$$

where I(z) is an indicator function that equals 1 if block *z* belongs to the surveillance area, and 0 otherwise. Then, *Z* is the updated block list, where *z* ranges from 1 to

$$[\left(n_{a}-1\right)\times \left(n_{b}-1\right)-Q].$$

By doing so, the model can approximate the actual shape of the surveillance area and select optimal sensor locations within the area.

##### Selection of Candidate Sensor Locations

The set of center points of blocks in *Z* is considered as the candidate sensor location. The coordinates of the candidate sensor location is represented by:

(7) $$C=\{\left(\alpha_{e},\beta_{e}\right)|e\in Z\},$$

where *C* is a set of center points of blocks in *Z*, and *e* is a potential sensor location. Since water blocks cannot be selected as sensor locations, the center points of water blocks are excluded from *C* to ensure they are not considered as potential sensor locations.

##### Sensor Block Allocation

We compute the Euclidean distance

(8) $$d_{e,i}=\sqrt{{\left(\alpha_{e}-x_{i}\right)}^{2}+{\left(\beta_{e}-y_{i}\right)}^{2}},\;\forall e\in C,\forall i\in P^{M},$$

where $d_{e,i}$ is the distance between a sensor location e∈C and a point $i\in P^{M}$, where the associated coordinates of *e* are $\left(\alpha_{e},\beta_{e}\right)$. If a sensor of type *s* is placed at *e*, it can cover the points that are within its sensor range $R_{s}$. To determine the points within $R_{s}$ of a sensor of type *s* placed at *e*, we define $A_{e}^{s}$ as the set of coordinates of the points covered by the sensor, represented by:

(9) $$A_{e}^{s}=\left\{i\in P^{M}|d_{e,i}\leq R_{s}\right\},\;\forall e\in C,\forall s\in S.$$

To identify the blocks covered by each sensor at every potential location, we define $B_{e}^{s}$ as the set of blocks covered when a sensor of type s∈S is positioned at location *e*. Algorithm 1 iteratively checks whether each point *o* in the block *z* is in $A_{e}^{s}$. If any point in *z* is not in $A_{e}^{s}$, the algorithm sets the value of $A_{e}^{s}$ to False for that block *z*. If all the points in *z* are in $A_{e}^{s}$, the algorithm indicates that a sensor of type *s* at location *e* covers the block *z*.
**Algorithm 1** Computing the set of blocks covered by each sensor at each candidate location**for** each sensor of type *s* in *S* **do**      **for** each location *e* in *C* **do** initialize an empty set $B_{e}^{s}$;            **for** each block *z* in *Z* **do** all points in *z* are in $A_{e}^{s}$ = True;                  **for** each point *o* in block *z* in *Z* **do**                        **if** *o* is not in the set $A_{e}^{s}$ **then** all points in *z* are in $A_{e}^{s}$ = False; break;                        **if** all points in *z* are in $A_{e}^{s}$ **then** add *z* to the set $B_{e}^{s}$.

##### Probability of Detection and Misdetection of Sensors

To compute the average detection probability of a sensor placed at *e*, represented as $\zeta_{e}^{s}$, we consider the mean of all the probability values for sensor detection across blocks *z* in $B_{e}^{s}$ for a sensor of type *s* as follows:

(10) $$\zeta_{e}^{s}=\frac{1}{|B_{e}^{s}|}\sum_{z\in B_{e}^{s}}\omega_{z}^{s},\;\forall e\in C,\forall s\in S.$$

The probabilistic framework of sensor detection probability presents an important consideration to improve the detection of aircraft in the airspace by understanding the probability of detection. When an aircraft is present in the airspace, the likelihood that a sensor will detect it is known as the probability of detection. On the other hand, the probability of misdetection refers to the likelihood of not detecting an aircraft when it is actually present. An effective solution to address this issue is to ensure sufficient sensor coverage at a specific location by installing an adequate number of sensors. This ensures that at least one sensor can track aircraft that meet the minimum detection probability requirement, which is the minimum probability of detecting an aircraft that must be met by sensors to ensure reliable detection [50]. For example, if two sensors are placed at a location, each with a detection probability of 0.8, the probability that at least one of the sensors will detect the aircraft is 0.96. Hence, it is essential to consider the probability of misdetection, calculated by:

(11) $$\tau_{e}^{s}=1-\zeta_{e}^{s},\;\forall e\in C,\forall s\in S,$$

where $\tau_{e}^{s}$ is the probability of misdetection for a sensor of type *s* placed at location *e*.

Let $\kappa_{e}^{s}$ be the number of independent sensors of type *s* required to achieve a minimum required detection probability at location *e*, and let $\tau_{el}^{s}$ be the probability of misdetection of sensor *l* among the $\kappa_{e}^{s}$ sensors. Then, $\Upsilon_{e}^{s}$, as given in Equation (12), can be expressed as the probability that all sensors of type *s* placed at location *e* fail to detect AAM aircraft simultaneously.

(12) $$\Upsilon_{e}^{s}=\prod_{l=1}^{\kappa_{e}^{s}}\tau_{el}^{s},\;\;\forall e\in C,\forall s\in S.$$

If the probability of misdetection for each of the $\kappa_{e}^{s}$ sensors is the same, then $\Upsilon_{e}^{s}$ can be expressed as follows:

(13) $$\Upsilon_{e}^{s}={\tau_{e}^{s}}^{\left(\kappa_{e}^{s}\right)},\;\;\forall e\in C,\forall s\in S.$$

##### Minimum Required Detection Probability Constraint

To achieve the minimum required detection probability, denoted by *r*, we introduce a minimum required detection probability constraint given by:

(14) $$r=1-\Upsilon_{e}^{s},\;\;\forall e\in C,\forall s\in S.$$

Based on this constraint and Equation (13), we derive Equation (15) to determine the value of $\kappa_{e}^{s}$.

(15) $$\kappa_{e}^{s}=\frac{\log(1-r)}{\log(\tau_{e}^{s})},\;\;\forall e\in C,\forall s\in S.$$

##### Objective Function and Decision Variables

The objective of the SAND model is to determine the optimal locations for sensor placement and the number of required sensors such that it minimizes the function

(16) $$min\left(\theta \right)=\sum_{\forall s\in S}\sum_{e\in C}\lambda_{e}^{s}\kappa_{e}^{s}\delta^{s}\psi^{s},$$

where θ is the total sensor cost. This function depends on three parameters: (1) $\psi^{s}$, the cost per unit of a sensor of type *s*; (2) $\kappa_{e}^{s}$; and (3) $\delta^{s}$, the number of sensors needed for sensor type *s* to provide $360^{\circ }$ coverage at a location. The value of $\delta^{s}$ depends on the field of view of sensor type *s*. For example, if the sensors have a field of view of $90^{\circ }$, then four sensors of the same type are needed to be positioned at equal intervals around the location to provide full coverage. If the sensors have a wider or narrower field of view, fewer or more sensors may be needed to ensure complete coverage, respectively.

Additionally, θ also depends on a binary decision variable $\lambda_{e}^{s}$, represented as follows:

(17) $$\lambda_{e}^{s}=\left\{\begin{array}{cc} 1,\; & \mathrm{if}\;\mathrm{sensor}\;\mathrm{is}\;\mathrm{placed}\;\mathrm{at}\;e\in C,\mathrm{where}\;s\in S\\ 0,\; & \mathrm{otherwise}.\; \end{array}\right.$$

**Figure 4:**
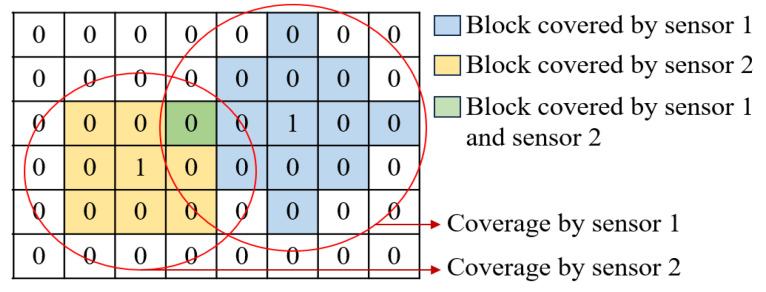
An example of a 6 × 8 mesh demonstrates how $\lambda_{e}^{s}$ determines the sensor locations and $\gamma_{z}$ the blocks covered by these sensors.

The value of $\lambda_{e}^{s}$ is 1 if a sensor of type *s* is placed at location *e* and 0 if no sensor is placed. For example, consider a surveillance area represented as a mesh with six rows, eight columns, and a total of 48 blocks, as demonstrated in Figure 4. The center points of these blocks serve as candidate sensor locations where sensors can potentially be placed. In this example, two of these blocks have $\lambda_{e}^{s}$ values of 1, indicating the placement of sensors at those locations. Conversely, the remaining blocks have $\lambda_{e}^{s}$ values of 0, indicating that no sensors are positioned at their center points. Therefore, the optimal number of sensors is two, and their optimal locations are the center points of the two blocks, where each of these center points is associated with specific GCS or PCS coordinates.

We introduce $\gamma_{z}$ as another binary decision variable, defined as follows:

(18) $$\gamma_{z}=\left\{\begin{array}{cc} 1,\; & \mathrm{if}\;\mathrm{block}\;z\in Z\;\mathrm{is}\;\mathrm{fully}\;\mathrm{covered}\;\mathrm{by}\;\mathrm{at}\;\mathrm{least}\;\mathrm{one}\;\mathrm{sensor}\\ 0,\; & \mathrm{otherwise}.\; \end{array}\right.$$

The value of $\gamma_{z}$ is 1 if the entire area of block *z* is covered by at least one sensor from any sensor type in *S* and 0 if the entire area of block *z* is not covered. In the example demonstrated in Figure 4, the red circles depict the coverage range of two sensors from different types. The colored blocks represent the areas covered by these sensors, each assigned a $\gamma_{z}$ value of 1. A block can be covered by one or multiple sensors; here, the blue and yellow blocks are fully covered by the first and second sensors, respectively, while the green block is covered by both sensors. The uncolored ones represent blocks that are not fully covered, each having a $\gamma_{z}$ value of 0.

##### Full Coverage Constraint

As the surveillance area must be fully covered by the network, we consider a full coverage constraint, as presented in (19).

(19) $$\sum_{e\in C,z\in B_{e}^{s}}\lambda_{e}^{s}\geq \gamma_{z},\;\;\forall z\in Z,\forall s\in S$$

This constraint ensures that each block *z* is fully covered by at least one sensor placed at *e*.

##### Homogeneous and Heterogeneous Sensor Networks

The SAND model can be implemented to build two distinct types of surveillance sensor networks, namely homogeneous and heterogeneous sensor networks. A homogeneous sensor network consists of only one type of sensor, while a heterogeneous sensor network is composed of various types of sensors. For example, if a homogeneous sensor network is built with radar, *S* = {Radar}, where *S* exclusively includes sensors that are radars and excludes sensors from other types. On the other hand, in a heterogeneous sensor network, for example, *S* = {ADS–B, Radar, Acoustic, Optical Camera}, the set *S* can include sensors of different types.

##### Assumptions

Several assumptions are incorporated into the SAND model. Firstly, we assume that there are no potential sensor obstructions posed by natural or human-made structures in the surveillance region. Secondly, we assume that all AAM flights take place within an altitude of 400 m, which is within the range of all the different sensor types considered. Thirdly, sensor detection performance does not depend on the precise aircraft paths. Fourthly, we assume that there is no effect of weather on sensor performance. We do not consider the effect of sensor failures on the AAM surveillance network. Lastly, we assume a uniform distribution of AAM flight operations across the analyzed region.

#### 3.1.4. Solution Algorithm

Following the methodology described in Section 3.1, the SAND model is implemented in Python 3 using the Gurobi Python API, and the model is solved to determine the optimal values of the $\lambda_{e}^{s}$ and $\gamma_{z}$ variables using the Gurobi 10.0.1 × 64 Linux on a computer with a 3.00 GHz × 36 Intel^®^ Core™ i9-10980XE processor and 128 GiB of memory. The Gurobi optimizer performs a branch-and-bound search to find a global solution. This is a systematic technique for solving optimization problems by recursively partitioning the space into smaller branches and then solving each branch independently. To convert the GCS coordinates into PCS coordinates for modeling the surveillance area, we use the ’pyproj’ Python package, which can conduct geodetic calculations and cartographic transformations [56].

### 3.2. Low Altitude Surveillance Information Clearinghouse Features and Functionalities

The surveillance data generated by the optimized surveillance sensor network must be safely stored and processed, taking into account the stakeholder preferences and expectations of LASIC features and functionalities. To determine these preferences and expectations, a survey of AAM stakeholders was carried out. Based on the survey, expected LASIC features include access to real-time coverage maps, live surveillance feeds, offline archival surveillance data, and support for querying and analyzing surveillance data. The relevant functional and performance requirements of LASIC—namely, surveillance interface standards, sensor data sizes, and ping rate—are determined based on a system requirements study of LASIC.

The surveillance data can be processed either on cloud- or locally owned servers. Given the features of LASIC, a cloud-based server is considered to be more suitable for hosting the surveillance data of LASIC due to the following reasons. Firstly, local servers require a large amount of time and effort to set up and maintain it. They also require a lot of space and expensive hardware. On the other hand, cloud-computing servers can be a cost-effective solution for LASIC, as they eliminate the need to invest in expensive hardware and infrastructure. Instead, the LASIC operator (e.g., a given state’s department of transportation) would pay only for the resources they use in the cloud, which can help build a more cost-effective LASIC in the long run by reducing operational expenses and avoiding the capital expenditures associated with maintaining and upgrading local servers. Secondly, cloud-computing servers provide a higher level of security compared to local servers, as cloud-computing servers invest heavily in security measures such as firewalls, encryption, and intrusion detection systems. The data gathered from surveillance must be protected in LASIC from unauthorized access, cyber threats, and breaches to ensure the privacy of the system. Any unauthorized access to the data can result in potential harm to the system and damage to the reputation of the LASIC program. The advanced security measures of cloud-computing servers can ensure the confidentiality and integrity of the AAM surveillance network and LASIC, which is essential to maintaining the trust of their constituents and regulatory compliance. Lastly, cloud-computing servers offer the advantage of being able to easily scale up or down based on changing demands. This is especially crucial in the context of LASIC and AAM surveillance, as the demand for advanced air mobility is rapidly increasing. As more AAM vehicles take to the skies, the amount of data generated by these vehicles will also increase, and the computing resources required to process and store this data will need to be adjusted accordingly. Conversely, local servers have a fixed number of resources and require additional hardware investments to accommodate additional demands. This can be a significant disadvantage for LASIC operators, who may need to invest in new hardware to accommodate increased demand, resulting in higher upfront costs.

Given the benefits discussed above, we deem a cloud-based server to be the most suitable host for LASIC. The cost of operating the server or utilizing cloud-computing services depends on AAM traffic projections, surveillance data generated in a specific area, surveillance data types, interface standards, data sizes, ping rate, cloud components, and pricing policies, which are discussed next.

#### 3.2.1. Surveillance Data Types and Sizes

The cost of cloud-computing services is associated with the types and sizes of surveillance data, as cloud vendors generally employ a billing model based on the number of data held or processed in the cloud infrastructure. As such, larger volumes of data processed or stored in the cloud result in higher cloud-computing costs. The type and size of surveillance data generated by the sensors in the AAM surveillance network depend on the type of service level provided by LASIC and the surveillance data interface standard. The three possible service levels that can be provided to subscribers of LASIC are informational only, radio location quality, and radio navigation quality. Of all the service levels, the radio navigation quality requires stringent data requirements that specify strict and precise data specifications, as it supports tactical deconfliction services. This is necessary to provide highly accurate navigation and positioning information, which is crucial for avoiding collisions and ensuring effective coordination between AAM aircraft during flight. To specify the interface standard for LASIC, the All-purpose STructured EUROCONTROL Surveillance Information eXchange (ASTERIX), as mentioned in [57,58,59], is used in this study, which is a collection of interface definitions and documentation outlining the data format standards used for transmitting a range of surveillance data.

The size of the total yearly surveillance data generated in a given area is determined based on several factors, including the projected yearly flight hours of AAM traffic in the area for potential use cases, such as passenger and cargo transportation, bridge inspections, small package delivery, and medical item delivery. We obtained the data of yearly estimated AAM passenger and cargo traffic from [60] and conducted forecasting to estimate the demand for other AAM use cases in [2]. Our assumption for the distribution of AAM flight hours over the given area is uniform. The size of the total yearly surveillance data also takes into account the size of the surveillance data messages, which are the packets of data generated by the sensors used for surveillance. These packets may contain information such as images, video, location data, and other types of sensor data. Additionally, the size of the total yearly surveillance data is calculated based on a ping rate of 1 Hz, which refers to the frequency at which the surveillance data is transmitted. A data ping rate of at least 1 Hz is necessary to provide real-time surveillance generated by the sensors. The AAM traffic is considered to comprise three main types of aircraft: cooperative manned aircraft, cooperative uncrewed aircraft, and non-cooperative aircraft. The surveillance message sizes associated with these types of aircraft are computed using their corresponding interface standards as defined in ASTERIX [57,58,59], which are listed in Table 3.

#### 3.2.2. Cloud Components

The cloud-computing cost is determined by the pricing policies of the cloud server chosen to host LASIC and the cloud components needed to enable the desired real-time and offline LASIC features and functionalities. The Microsoft Azure Web cloud-computing services are considered in this study to estimate the cloud-computing cost. Microsoft Azure provides a range of cloud-based services that can be utilized to create a platform for real-time analysis of live surveillance data. It can be used to continuously ingest and process LASIC data in near-real time and store the data for data archival, dissemination, querying, and analytics.

**Figure 5:**
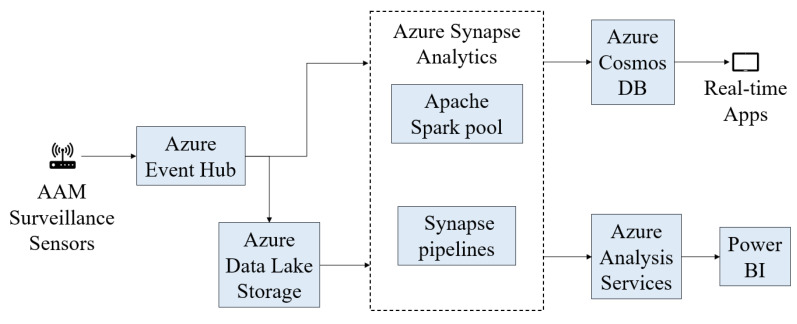
A flowchart showing the connections of the cloud components of LASIC.

A cloud-computing architecture capable of real-time analytics on big data would need to be created to enable the data flow through LASIC. The cloud-computing architecture would consist of six components: (1) Azure Event Hub, (2) Azure Synapse Analytics, (3) Azure Data Lake Storage, (4) Azure Cosmos Database (DB), (5) Azure Analysis Services, and (6) Power BI [61]. The cloud components of LASIC are depicted in Figure 5. The Azure Event Hub is a big data streaming platform and event ingestion service where millions of data units can be received and processed in a single second [62]. It can be used to easily ingest live streaming data from the AAM surveillance sensors. Then, Azure Synapse Analytics can be used to transform and store data that have been provided to the Azure Event Hub, respectively. Azure Synapse Analytics is an analytics service that combines data integration, enterprise data warehousing, and big data analytics [63]. For large-scale access and movement of surveillance data, Azure Synapse Analytics would require the use of Apache Spark pool and Synapse pipelines. These components can be used for data cleaning, transforming, and analyzing; and can enable the use of Python, Scala, or .NET, and scalable ML techniques to derive deeper insights from LASIC data. Azure Data Lake Storage allows massively scalable and secure data lake functionality built on Azure Blob Storage [64], which is needed to store the LASIC data. To provide access to the intended LASIC data to subscribers through real-time apps, data would need to be transferred from Apache Spark pools to Azure Cosmos DB [65]. Analytic dashboards and embedded reports on LASIC data can be created using Azure Analysis Services and Power BI for use by the LASIC operator and subscribers [66,67].

### 3.3. Cost–Benefit Analysis of Low Altitude Surveillance Information Clearinghouse

To assess and justify the worth of investing in an AAM surveillance network and LASIC, a cost–benefit analysis is needed, as mentioned in Section 1. Hence, we conducted the cost–benefit analysis, which involves identifying and estimating the potential costs and benefits associated with this infrastructure. The findings of the first two parts are used to perform the analysis, as shown in Figure 3. The cost and benefit factors identified and considered to be significant in this analysis are (1) the surveillance sensor cost (cost factor 1), (2) the cloud-computing cost (cost factor 2), and (3) revenue generated from a LASIC subscription (benefit factor), which are discussed more in this section. Using the estimates of the cost and benefit factors, the NPV is calculated, which is necessary to measure the future return on investment expected from an investment in a project in terms of today’s dollars. The NPV metric takes into account the time value of money and future cash flows, which is further discussed in Section 3.3.4. As there is an uncertainty associated with some of the key parameters involved in the analysis—namely the subscription fees, number of initial subscribers, terrain-based sensor detection probabilities, and the minimum required detection probability—a sensitivity analysis was performed to evaluate the effect of these parameters on the NPV generated.

#### 3.3.1. Cost Factor 1: Surveillance Sensor Cost

The first cost factor is the surveillance sensor cost, which is the total cost incurred to build the surveillance network in a given area. This cost factor depends on the number of sensors needed to obtain the intended coverage over a given region and the price of the sensors. The required number of sensors is evaluated by implementing the SAND model described in Section 3.1.3 for six sensor types mentioned in Section 3.1.1. In this analysis, we consider the capital for purchasing and installing sensors to build the surveillance network that will be invested once in 2023.

#### 3.3.2. Cost Factor 2: Cloud Computing Cost

As discussed in Section 3.2, opting to host LASIC on a cloud server incurs an annual cloud-computing cost that constitutes the second cost factor throughout the analysis period. To estimate this cost, we refer to Microsoft Azure’s pricing policies, as outlined in [68], which take into account the number of surveillance data published and received by LASIC. The amount of surveillance data generated in a particular area can vary depending on factors such as an AAM traffic projection, surveillance data type, interface standard, data size, and ping rate.

#### 3.3.3. Benefit Factor

The survey responses reveal a willingness to pay by potential subscribers of LASIC for the services offered by it. The range of subscription fees that potential subscribers are willing to pay for the services expected to be offered by LASIC is found to be USD 100–400 dollars. This informs the computation of the benefit factor considered in this study and the revenue generated from LASIC. The potential subscribers of LASIC include parcel and cargo delivery operators, medical item delivery companies, air taxi operators, infrastructure inspection companies, airspace service providers, state penitentiaries, law enforcement agencies, correctional facilities, and municipalities. The number of potential subscribers in the various years of the analysis period is estimated based on global and US AAM market growth rates reported in AAM market studies, such as [69,70,71].

#### 3.3.4. Net Present Value

To evaluate the financial viability of LASIC, its NPV over the analysis period needs to be computed and analyzed.

(20) $$NPV_{t}=\frac{C_{t}^{P}-C_{t}^{N}}{{\left(1+\chi \right)}^{t}},\;\;\forall t\in \left\{0,1,...,10\right\}$$

This represents the estimated total value of all future cash flows generated by an investment over the lifetime of the project or a given analysis period, where $C_{t}^{P}$ and $C_{t}^{N}$ represent the positive cash flow and negative cash flow in year *t*, respectively [72,73,74]. In this analysis, the yearly NPV calculation of the AAM surveillance network and LASIC is carried out based on the difference between the revenue generated (positive cash flow) and the costs associated with the AAM surveillance network and LASIC (negative cash flow). A discount factor is considered to account for the time value of money, which reflects the idea that a dollar received in the future is worth less than a dollar received today. We considered a cash flow over a 10-year horizon, discounting at 10% (the discount rate χ) for this infrastructure project [75,76,77]. A positive NPV at the end of the analysis period implies that the expected revenue from the investment exceeds the projected costs, and thus, the investment is considered profitable. Conversely, a negative NPV suggests that the investment would result in a net loss.

## 4. Results

The applicability of the proposed model and solution approaches are demonstrated through their application in several numerical experiments in this section. To undertake the experiments, we consider the six major cities of Ohio (SMCO): Columbus, Cleveland, Cincinnati, Akron, Toledo, and Dayton. The formation of SMCO is predicated on the finding of significant demand potential for AAM use cases in those cities considering socioeconomic factors, such as population, population density, gross domestic product, median per capita income, cost of living, total area, cities in motion index, human capital, etc. [60].

### 4.1. Experimental Setup

In this section, we provide details of the values related to the sensors and surveillance area that we consider in our experimental setup for running the experiments.

#### 4.1.1. Sensors

In this study, one real-world sensor model is considered for each of the sensor types discussed in Section 3.1.1. The Echo Guard radar is considered for the radar sensor type. It is a top-tier 4D radar with an easy user interface that is easily adaptable to site and mission requirements for high-performance ground-based detect-and-avoid [21]. We consider CamelCase pingStation3 V2.4.43 as an ADS–B frequency ground receiver for our analysis. It is a networkable weatherproof 978/1090 MHz ADS–B receiver that includes GPS and an antenna, with power and data provided by a single power-over-Ethernet network cable connected to a LAN [18]. We consider DroneScout as the (direct/broadcast) remote ID receiver, which can receive remote ID signals sent from aircraft [20]. The Dedrone RF-360 is considered in this study for the RF sensor type. It is a passive, network-attached radio sensor used for the detection, classification, and localization (geolocation) of aircraft and their remote controls [19]. The OptiNav Drone Hound system is considered the acoustic sensor type in our study. It is an acoustic sensor that has been designed to detect, identify, and track sUAS. Unlike other sensors, it does not rely on electromagnetic emissions from the sUAS [22]. We consider the Q6225-LE PTZ Network Camera from Axis Communications in our analysis for the optical camera sensor type [78].

The *R*, ψ, and δ of the six selected sensors from their corresponding sensor types in the input set *S* of the SAND model are listed in Table 4. The sensor types listed vary in terms of their range and cost. Radar has the highest range of 321.87 km, provided by the ADS–B sensor. Remote ID and RF sensors have ranges of 5.02 km and 4.99 km, respectively, while acoustic and optical camera sensors have much shorter ranges of 0.5 km and 0.4 km, respectively. Radar and RF sensors are generally the most expensive, with one of the sensors costing around USD 35,000—the other sensors in the table range in price from USD 1100 to USD 9000.

#### 4.1.2. Surveillance Area

In the experimental setup for modeling a surveillance area, we considered the following factors to execute the SAND model. As mentioned in Section 3.1.3, $L/\sqrt{2}$ should be greater than ρ. Therefore, we consider *L* as 0.3 km, which is less than 0.4$\sqrt{2}$ km, where 0.4 km is the lowest value of the range among all sensor types *S* (refer to Table 4). This ensures that all blocks are covered by the sensors and no surveillance area is left uncovered. Hence, to run the SAND model for Columbus, Cleveland, Cincinnati, Toledo, Akron, and Dayton, we generated 130 × 126, 77 × 96, 58 × 77, 57 × 72, 65 × 62, and 54 × 60 blocks, respectively.

A higher minimum required detection probability value would need to be considered for security-sensitive areas to ensure a higher level of detection and identification accuracy. We considered the value of *r* as 0.98 in our study, which means that the system aims to detect and identify targets with a probability of at least 0.98. For considering the effect of terrains on the probability of detection of a sensor, the terrains of the area are divided into five major types of *T*: (1) open, (2) water, (3) neighborhood or residential area, (4) hill, and (5) busy commercial area or downtown. The Google Maps platform is utilized to observe and determine the terrain type of each block z∈Z. $T_{z}$ is obtained by reference to the terrain classification of the *z*-th block, as recorded in the list *T*. The probability values in the $\omega_{T}^{s}$ matrix, as given in Table 5, are approximated based on the approach reported in [79,80,81]. By analyzing the values presented in the table, it is evident that the detection probabilities of the sensors tend to decrease as the terrain type changes, which aligns with the discussion in Section 3.1.2. The probability of detection is the highest for open terrains, followed by water and neighborhood, whereas it is comparatively lower for hill and commercial areas. Additionally, the values indicate that the detection probabilities for radar and the ADS–B sensor are relatively higher than other sensor types, regardless of the terrain type. Conversely, the acoustic sensor has the lowest detection probability among all sensor types and terrain types. These observations emphasize the significance of selecting an appropriate sensor type and its detection probability when designing a sensor network for a given area with varying terrain types.

**Figure 6:**
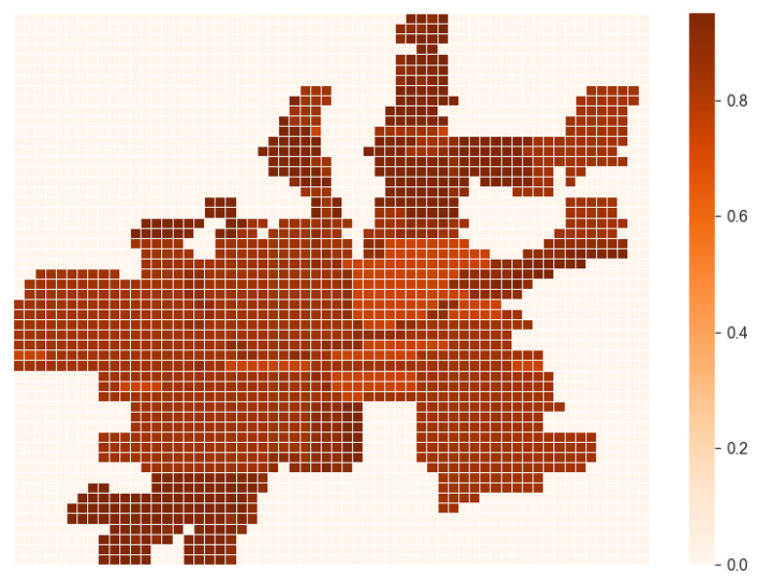
A heatmap of the probability of detection of a radar based on terrain types of 3240 blocks in Dayton.

To demonstrate the dependence of a sensor’s detection probability on the terrain type, Figure 6 presents a heatmap showing the probability of detecting radar across the various terrain types in Dayton. The colored bar on the right side of the figure shows the scale of detection’s probability, where the off-white color refers to a probability of zero and the darkest orange color refers to a probability of one. Note that the detection probability of sensors in blocks outside the area, where I(z)=0, is set to zero. Hence, the off-white color refers to blocks that do not belong to Dayton, and the orange colors, on the other hand, represent blocks within the area. By setting the detection probability of sensors in blocks outside the area to zero, the analysis is focused on the sensors within the area of interest. This allows for a more precise evaluation of the sensor network’s effectiveness in the designated area.

**Figure 7:**
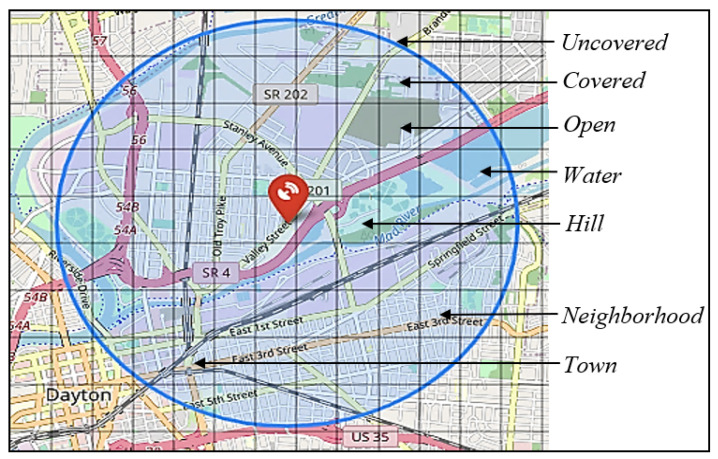
Block selection overview-classification of blocks by sensor coverage and terrain type.

Considering the sensor detection probabilities in different terrain types, Figure 7 presents an overview of the selection process that the SAND model uses to select blocks within a sensor range. The red marker refers to the location where a sensor is placed, and the blue circle shows the area within its range. Blocks that do not have all four corner points inside the blue circle are classified as the “Uncovered” blocks (i.e., uncovered by the sensor range), whereas the blocks with all four corner points inside the blue circle are classified as the “Covered” blocks. Each of the covered blocks can belong to any of the five terrain types in *T*, as mentioned in Figure 7 in italic font.

Following the methodology described in Section 3.3, determining the number of sensors needed and their locations using the SAND model, the revenue, costs, and the NPV of the AAM surveillance network and LASIC are computed for the numerical experiments. Before discussing the homogeneous and heterogeneous sensor placement analyses, we address the analysis of the revenue and cloud-computing cost since they are the same for both experiments. The NPVs of the different sensor types are compared with each other in terms of two criteria: (1) the number of years required to reach the break-even point and (2) the estimated NPV in the final year of the analysis period. Given the uncertainty of the AAM market, a sensitivity analysis was conducted to examine how the NPV responds to changes in the key market parameters—namely, the yearly number of subscribers and the subscription fees. The results are presented in the following subsections.

### 4.2. Revenue and Cloud-Computing Cost Analysis

The total yearly revenues generated by the AAM surveillance network and LASIC are determined by two factors: the yearly number of subscribers and the monthly subscription fee of LASIC. It does not depend on the sensor type used in the surveillance network, provided that complete coverage is present across SMCO. Hence, for all sensor types, the revenue generated is the same. We estimated the number of subscribers for LASIC in 2024 to be 100 based on the existing number of sUAS operators, air taxi operators, infrastructure inspection companies, medical item delivery companies, state penitentiaries, law enforcement agencies, correctional facilities, and municipalities in SMCO. Then, we studied the compound annual growth rate (CAGR) of the market size of AAM to estimate the number of yearly subscribers of LASIC over the analysis period based on the reports available on global and US AAM market growth [69,70,71,82]. Because of the evolving nature of the AAM market and its inherent uncertainty, we incorporated a CAGR range of 10% to 20% in our study based on the values reported in the AAM market growth studies instead of relying on a fixed value of CAGR. Here, the lower limit of 10% signifies the conservative estimate, while the upper limit of 20% represents the optimistic estimate.

**Figure 8:**
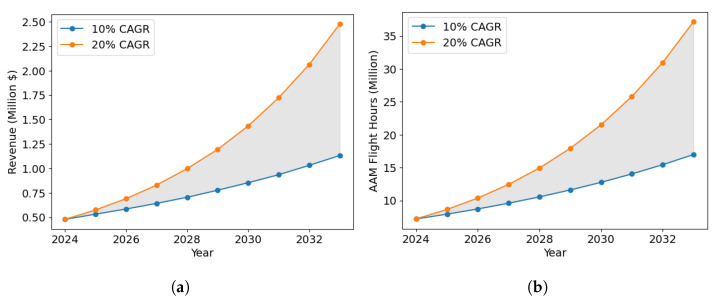
Yearly revenues and yearly projected AAM traffic. (**a**) Yearly revenues generated by AAM surveillance network and LASIC. (**b**) Yearly projected AAM traffic in Ohio.

Another factor that affects the revenue is the monthly subscription fee, which ranges between USD 100 and USD 400, as mentioned in Section 3.3.3. For the revenue analysis, we assume the fee to be USD 400 and later vared it during the sensitivity analysis to analyze cases where the fee is less than USD 400. The yearly revenues generated by the AAM surveillance network and LASIC in SMCO, with an initial number of subscribers of 100 and a fixed subscription fee of USD 400, are depicted in Figure 8a. As the number of subscribers increases over the years, the revenue grows proportionally. The grey shaded region signifies the potential revenue outcome that falls between the projected revenues at a 10% CAGR and those at a 20% CAGR. It also highlights how the growth rate significantly impacts the range of revenue projections over time. In the case of a 10% CAGR, the revenue is expected to begin at USD 0.48 million and increase gradually over the years, reaching approximately USD 1.032 million in 2033. On the other hand, with a more optimistic 20% CAGR, the revenue starts at the same initial value of USD 0.48 million but experiences a more rapid growth, reaching a potential high of USD 2.064 million by 2033.

**Figure 9:**
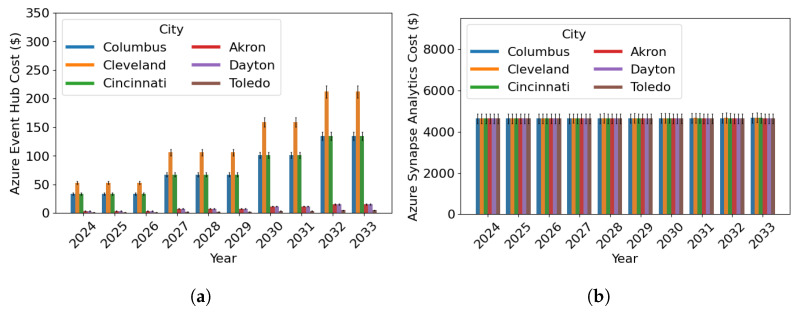

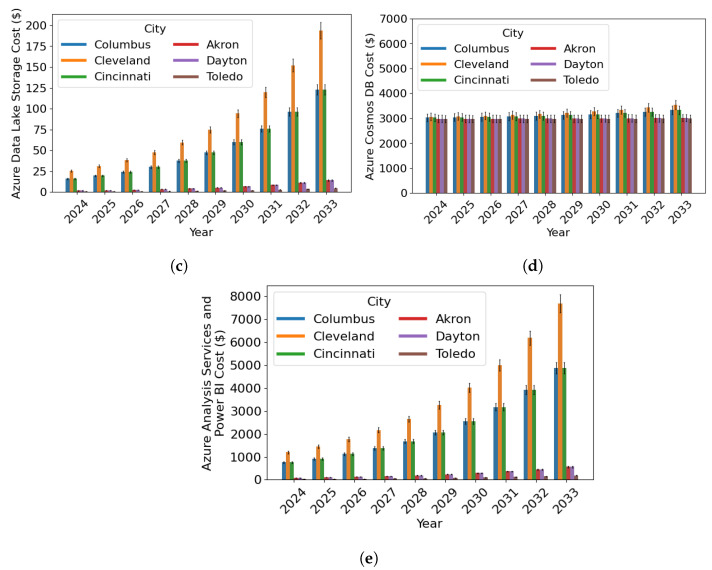
Costs of different cloud components in cloud computing. (**a**) Azure Event Hub cost. (**b**) Azure Synapse Analytics cost. (**c**) Azure Data Lake Storage cost. (**d**) Azure Cosmos DB cost. (**e**) Azure Analysis Services and Azure Power BI cost.

The cost of cloud computing, according to the Microsoft Azure pricing policies, depends on the projected amount of surveillance data generated in each city, which in turn depends on the projected AAM traffic in each city, as presented in Figure 8b. The range of CAGR values of the AAM market size considered accounts for the uncertainty in AAM flight hours. This uncertainty range subsequently influences the cloud-computing cost, as illustrated in the figures through the inclusion of error bars. These error bars serve to indicate that the associated cost is estimated to fall within the limits defined by the bar. The ten-year cloud-computing cost breakdown for each cloud component and each city within SMCO is illustrated in Figure 9. The Azure Event Hub and Azure Data Lake Storage have the two lowest costs among all the components. The Azure Event Hub operates on a tiered pricing model, where the cost of the service varies based on the level of usage of surveillance data by a subscriber. The cost is relatively low in the first few years, as the surveillance data and the number of subscribers are initially low, and the cost increases in steps as the surveillance data and number of subscribers increases. When the Azure Event Hub usage reaches a defined threshold, the cost climbs to a higher level, and this pattern repeats for each subsequent tier, creating a step function of the cost with respect to usage. The costs for Azure Event Hub and Azure Data Lake Storage increase with the amount of incoming data ingested into the hub and stored in the Data Lake, respectively. Additionally, the frequency of data access also influences the rise in cost, with higher amounts of access due to an increasing number of subscribers, which lead to an increase in cost in successive years. The Azure Analysis Services and Azure Power BI costs increase with time, commensurate with the projected increase in the amount of data stored, number of queries run, and number of users accessing the services. Lastly, the pricing of Azure Synapse Analytics and Azure Cosmos DB includes both a yearly fixed cost and a yearly variable cost. The yearly fixed cost is associated with the provisioning of virtual machines, storage, and other necessary resources to operate the services. The yearly variable cost depends on the amount of data processed in LASIC. As the yearly fixed cost is much higher than the yearly variable cost, the Azure Synapse Analytics and Azure Cosmos DB costs are nearly constant, increasing slightly over the years. Across all cities, the cloud-computing cost associated with ingesting, storing, and analyzing the surveillance data generated in Cleveland is the highest, as it has the highest air traffic demand forecast across SMCO and hence produces the largest amount of surveillance data, whereas for Toledo, the cost is the lowest, as it generates the lowest air traffic demand forecast, and hence the lowest amount of data.

### 4.3. Homogeneous Sensor Placement Analysis

In the homogeneous sensor placement analysis, the surveillance network across SMCO is considered to be built using one sensor type instead of a mix of sensor types. This allows for a more in-depth analysis of each individual sensor type’s suitability for AAM surveillance and capability to produce the NPV over the analysis period.

**Figure 10:**
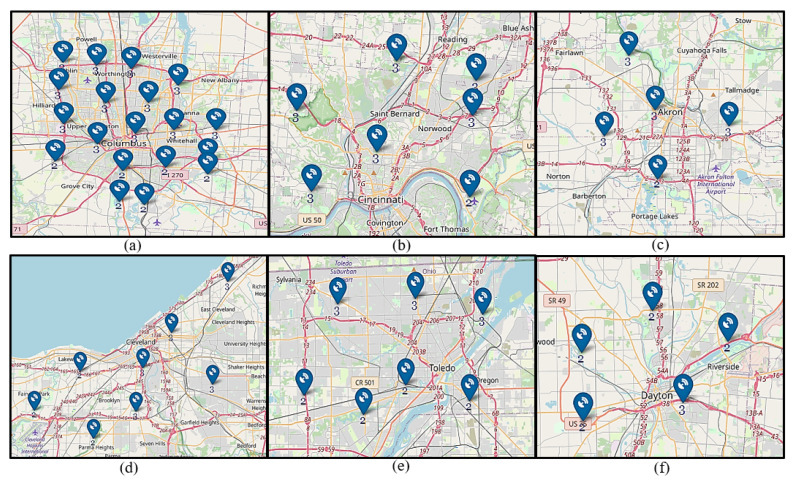
Optimal locations of RF sensors (the blue markers) in six cities: (**a**) Columbus, (**b**) Cincinnati, (**c**) Akron, (**d**) Cleveland, (**e**) Toledo, and (**f**) Dayton.

For each sensor type, the optimal location and number of sensors required to build the homogeneous surveillance network at a minimum sensor cost in SMCO are determined using the SAND model. The optimal locations for RF sensors in the surveillance network across SMCO are shown in Figure 10, where the blue markers represent the locations of the RF sensors. The sensors are strategically placed to ensure that all blocks within the sensor range are covered while minimizing the overlapping region to reduce the cost of the sensor network. The distribution of sensor locations varies for each city based on factors such as the city shape and area, terrain type, and probability of detection of each sensor type based on the terrain types. These factors also affect the optimal location and number of sensors required to provide adequate coverage of the city and determine the total sensor cost. For example, Columbus can be approximated as having a circular shape, while Cleveland is wider than it is long, and Columbus has a larger area compared to cities like Akron and Dayton. Another instance is Toledo, which has a greater number of water bodies compared to Columbus, while Cincinnati has more hilly terrain. These variations in shape and terrain result in different sensor distributions in the network.

The number of sensors required varies significantly depending on the city and the type of sensor used, as shown in Table 6. For any given sensor type, the number of sensors required to cover a given area increases with the area of the city. Among SMCO, Columbus requires the largest number of sensors, as it has the largest area, and Dayton the smallest, as it has the smallest area. In addition, the range of the sensors used also affects the number of sensors required. Radar sensors, for example, typically have a longer range than optical cameras (refer to Table 4), which means that fewer radar sensors are needed to cover the same city compared to using more optical cameras. Different cities have varying proportions of terrain types (refer to Figure 10). The detection probabilities of sensors on different terrain types are affected by the sensor type used (refer to Table 5), which in turn affects the number of sensors required. For instance, acoustic sensors have a lower detection probability than other sensor types when placed on a block with hilly terrain. As a result, cities with hilly terrain, such as Cincinnati, require more sensors to cover the terrain than cities like Cleveland with relatively less hilly terrain. Therefore, the number of sensors needed in Cincinnati is higher than the number of sensors needed in Cleveland for acoustic sensors, even though Cleveland is larger in size compared to Cincinnati. Moreover, the field of view varies with the sensor type, and for a limited field of view, more sensors are required to ensure a complete 360° view. For instance, although the ranges of acoustic sensors and optical cameras are similar, acoustic sensors have a lower detection probability compared to optical cameras. Thus, the number of sensors required for acoustic sensors to cover an area should be higher than that required for optical cameras. However, due to the higher value of δ for optical cameras compared to acoustic sensors (refer to Table 4), the number of sensors required becomes higher for optical cameras than for acoustic sensors.

**Figure 11:**
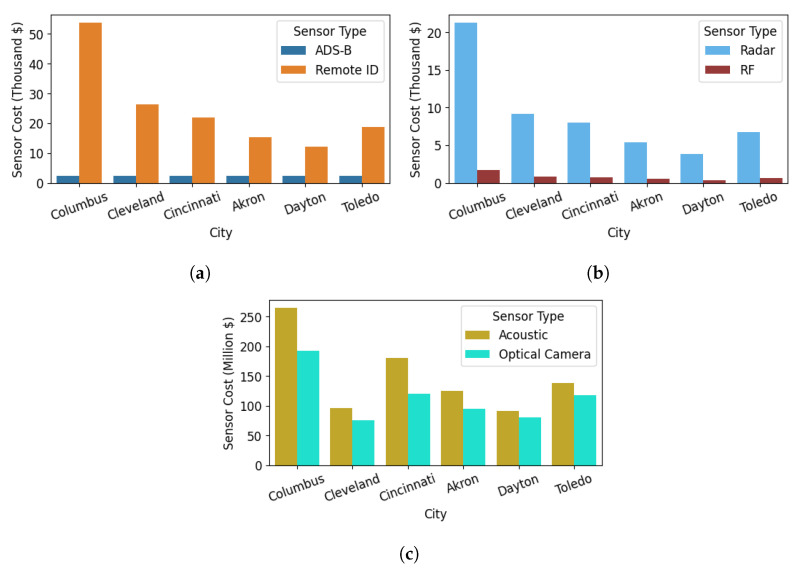
City-wise sensor costs for different sensor types. (**a**) Sensor costs of ADS–B and remote ID. (**b**) Sensor costs of radar and RF. (**c**) Sensor costs of acoustic and optical camera.

Based on the unit price of each sensor type and the number of sensors required for a sensor type for each city, the SAND model determines the city-wise sensor cost of all sensor types. The sensor costs of all cities are presented in Figure 11. The different sensor types listed in ascending order of sensor cost are ADS–B, remote ID, RF, radar, optical camera, and acoustic. Among SMCOs, Columbus requires the highest sensor cost for all sensor types, while Dayton has the lowest. The sensor costs for ADS–B and remote ID sensor types are much lower compared to the other sensor types, as they require fewer sensors and have lower unit prices. At the other end of the sensor cost spectrum are optical cameras and acoustic sensor types. Though the unit prices of acoustic sensors and optical cameras are cheap, a large number of sensors are required for each to cover the SMCO, as mentioned above, resulting in a very high total sensor cost. While the number of sensors required for the acoustic sensor type is less than for optical cameras, the total sensor cost for acoustic sensors is higher than for optical cameras due to the lower unit price of optical cameras.

**Figure 12:**
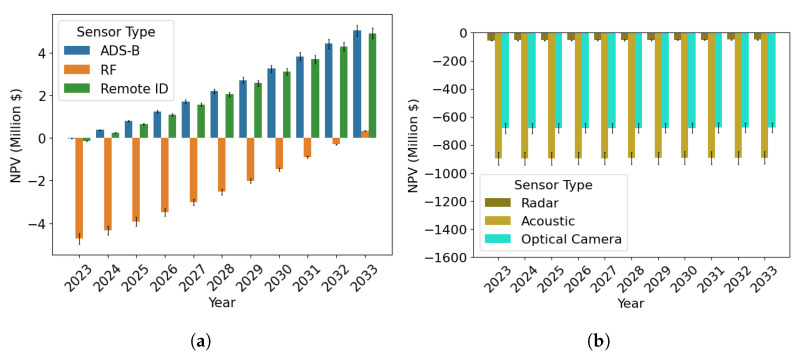
Yearly NPVs of six sensor types. (**a**) Yearly NPVs of ADS–B, RF, and remote ID. (**b**) Yearly NPVs of radar, acoustic, and optical camera.

Utilizing the total sensor cost for the SMCO presented in this section, as well as the revenue and cloud-computing cost for the SMCO detailed in Section 4.2, the NPV is calculated. The yearly NPVs associated with all sensor types are presented in Figure 12, where the length of an error bar for a sensor type in a given year, extending on either side of its central NPV value, indicates the range within which the NPV for that sensor type in that year is expected to fall. For all sensor types, the steady increase in the NPV with time is fueled by the increase in yearly revenues generated from the subscription fees. This NPV growth is less noticeable for the radar, optical camera, and acoustic sensor types, as they have high initial sensor costs. ADS–B, remote ID, and RF sensor types generate positive NPVs within the analysis period. ADS–B generates the largest NPV, followed closely by remote ID, while RF brings the third-largest NPV. These sensor types lead the NPV race because they have lower unit prices and higher ranges, thus requiring fewer sensors to cover a city and, hence have lower sensor costs. Both the ADS–B and remote ID sensor types will quickly reach the BEP in 2024. Their projected NPVs reach around USD 5.04 million and USD 4.90 million in the final year of the analysis period, as illustrated in Figure 12a. The RF sensor type takes longer to reach the BEP, gaining a positive NPV of approximately USD 3.29 million in 2033. On the other hand, as shown in Figure 12b, the projected yearly NPVs for the radar, acoustic, and optical camera sensor types feature negative NPVs over the 10-year analysis period due to their high initial sensor costs.

Policy recommendation: As discussed previously in Section 3.1.1, each sensor type can detect and track cooperative and/or non-cooperative aircraft flying in low-altitude airspace. Radar, RF, acoustic, and optical cameras are capable of tracking both types of aircraft, while ADS–B and remote ID can only track cooperative aircraft. Based on the NPV, if tracking only cooperative aircraft is sufficient, then ADS–B and remote ID sensor types are recommended, as they are the most financially viable sensor types for the AAM surveillance network and LASIC. If tracking both cooperative and non-cooperative aircraft, especially those flying over penitentiaries and other restricted areas, is a requirement, then RF is the most profitable sensor type.

### 4.4. Heterogeneous Sensor Placement Analysis

The heterogeneous sensor placement analysis aims to investigate the network composition and costs associated with using a combination of sensors of different types rather than selecting sensors of just one type. The SAND model identifies the optimal sensor locations of the assorted sensor types to build the AAM surveillance sensor network across SMCO. To conduct an experiment on the heterogeneous sensor placement analysis, we considered providing coverage to sensitive locations within SMCO, such as penitentiaries, police stations, and airports, where detecting both cooperative and non-cooperative aircraft is equally important. Since radar, RF, acoustic, and optical camera sensor types can detect both types of aircraft, we initially considered the set of sensors as *S* = {Radar, RF, Acoustic, Optical Camera}. However, we find that only the RF sensor type is selected from this set, as it dominates other sensor types and generates the same results as the RF sensor type in the homogeneous case (refer to Section 4.3). This is due to the RF sensor type having a larger range, higher detection probability on all terrain types, higher field of view (lower δ), and a lower unit price compared to other sensor types in the set. We then considered the set of sensors as *S* = {Radar, Acoustic, Optical Camera} to conduct the experiment again and generate further insights on the heterogeneous sensor placement analysis, which is given in this section.

The total number of sensors and sensor cost needed to place the sensors, as presented in Table 7 and Table 8, respectively, are compared between the two types of sensor placement: the homogeneous sensor network and the heterogeneous sensor network. For each city, the homogeneous cases show the values of the total number of sensors and the sensor cost of placing three individual sensor types (radar, acoustic, and optical camera), whereas the heterogeneous case shows the values for a mix of these sensor types. Comparing the values shows that heterogeneous sensor placement requires fewer sensors than acoustic sensors and optical cameras in a homogeneous sensor network but more sensors than radars in a homogeneous sensor network. For example, in Akron, the heterogeneous sensor network requires 155 sensors, as shown in Table 7, which is lower than the numbers required for acoustic and optical cameras, at 13,920 and 27,228, respectively, but higher than the number of sensors required for radars in the homogeneous sensor network, which is 153. However, the total cost of the sensors for setting up a heterogeneous sensor network is much lower than the separate costs for radar, optical cameras, and acoustic sensor types in the homogeneous case, which are USD 1.89 million, USD 13.53 million, and USD 31.56 million, respectively. The SAND model selects the optimal number and location of sensors to minimize the total cost of the sensors, even if it means using more sensors by replacing some radars with acoustic sensors. Although this results in an increased number of sensors, the use of lower-priced sensors reduces the total sensor cost, which is the objective of the SAND model.

The runtime (in seconds) of our algorithm applied to the homogeneous and heterogeneous sensor networks in various cities reveals a consistent pattern, as detailed in Table 9. In larger cities, where a higher number of blocks are required, the computation times are longer, whereas in smaller cities, which necessitate fewer blocks, the computation times are shorter. These runtimes are lower compared to those reported in other studies, as listed in Table 1.

**Figure 13:**
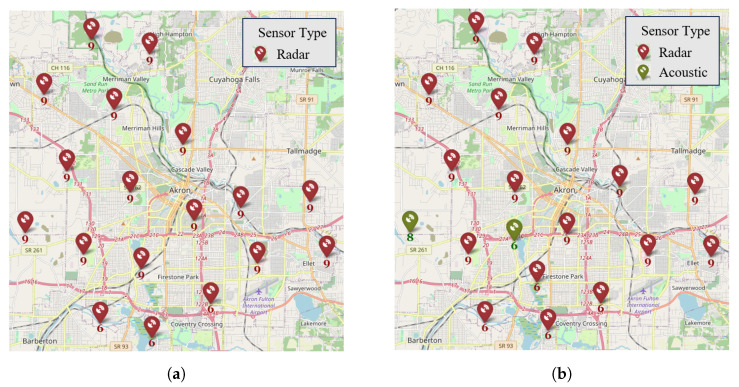
Optimal locations of sensors in Akron in homogeneous and heterogeneous sensor networks (texts below the markers show the number of sensors needed at the respective locations). (**a**) Optimal locations of sensors in homogeneous network. (**b**) Optimal locations of mixed sensors in heterogeneous network.

The SAND model reconfigures the number and location of sensors in the heterogeneous sensor network, as demonstrated in Figure 13, to optimize the total sensor cost more effectively. The figure shows the optimal sensor locations in both the homogeneous and heterogeneous sensor networks for the city of Akron, where red markers represent radar locations, green markers represent acoustic sensor locations, and the numbers below the markers indicate the number of sensors needed at each location. In the heterogeneous sensor network, an optimal combination of sensors with appropriate ranges is chosen to cover the given area, resulting in lower costs compared to the homogeneous sensor network. In other words, the ranges of the different sensor types are utilized effectively in the heterogeneous sensor network to reduce the sensor cost. For example, near the outer edges of the city and in small pockets within the city, sensors with a smaller range and lower unit price are placed, such as acoustic sensors, instead of sensors with a higher range and higher unit price, like radar, to minimize the sensor cost.

**Figure 14:**
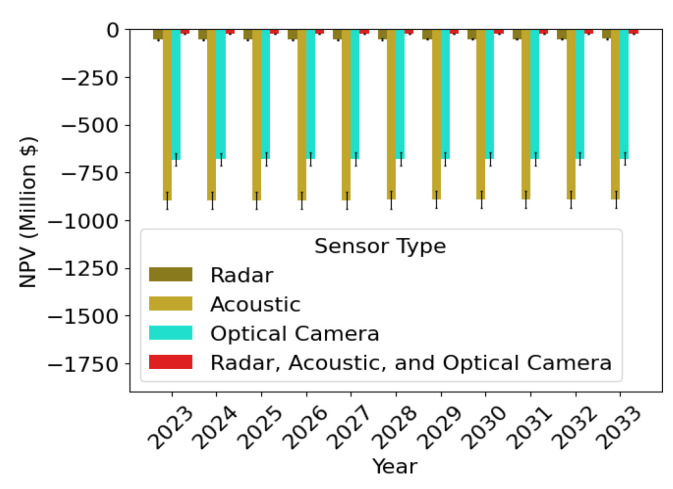
Comparison of yearly NPVs between the homogeneous and heterogeneous sensor networks.

We then calculated the NPV of the heterogeneous sensor network in SMCO by taking into account the revenue, cloud-computing cost, and total sensor cost. As shown in Figure 14, the NPV generated by the heterogeneous sensor network is higher than the NPVs generated by the respective homogeneous sensor networks for individual radar, acoustic sensors, and optical cameras. This is due to the lower sensor cost of the heterogeneous sensor network, which is discussed earlier in this section. The first three bars in the figure represent the homogeneous cases, while the last one represents the heterogeneous case with a mix of radar, acoustic sensors, and optical cameras. Although the NPVs for the radar, acoustic, and optical cameras mentioned in Section 4.3 are negative, there is a noticeable trend of increasing NPV values as we move from homogeneous cases to heterogeneous cases in all time periods. Therefore, we conclude that a heterogeneous sensor placement analysis can help generate more NPVs than a homogeneous sensor placement analysis.

Policy recommendation: The heterogeneous sensor network offers a lower sensor cost compared to a homogeneous sensor network, allowing for the identification of the optimal mix of sensors from a given set of sensor types. Therefore, among the two types of sensor placement, a heterogeneous sensor network is recommended when the right set of sensor types is selected, considering which types of aircraft need to be detected and tracked. By using heterogeneous sensor placement, it is possible to design a surveillance network with a minimum cost that installs sensor types that can track non-cooperative aircraft (e.g., RF) in security-sensitive areas (e.g., penitentiaries, law enforcement facilities, and correctional facilities), and sensor types that can track either cooperative or non-cooperative aircraft (e.g., ADS–B and remote ID) in nonsecurity-sensitive or general public areas. Other sensor placement constraints can also be enforced while designing a heterogeneous sensor network based on the requirements, preferences, and regulations of the government, AAM operators, and LASIC subscribers.

### 4.5. Sensitivity Analysis

Despite the growing interest in AAM, several unresolved obstacles and concerns exist, including the need for new widespread infrastructure to support AAM operations, such as vertiports, takeoff and landing sites, charging stations, air traffic control systems, airspace routes, and surveillance networks. Additionally, the current regulatory framework for air transportation is not designed for AAM, requiring new regulations and standards. Factors such as changes in consumer preferences, regulatory requirements, and technological advancements could all affect the adoption and growth of AAM services. Hence, in this section, a sensitivity analysis is conducted to examine the impact of the changes in parameters directly affecting the NPVs generated in our analysis, such as the LASIC subscription fee and the number of initial subscribers. We also conducted a sensitivity analysis for other key parameters that we are uncertain about due to the lack of verified data available, such as terrain-based sensor detection probabilities and the minimum required detection probability, to observe their impacts on respective outputs.

**Figure 15:**
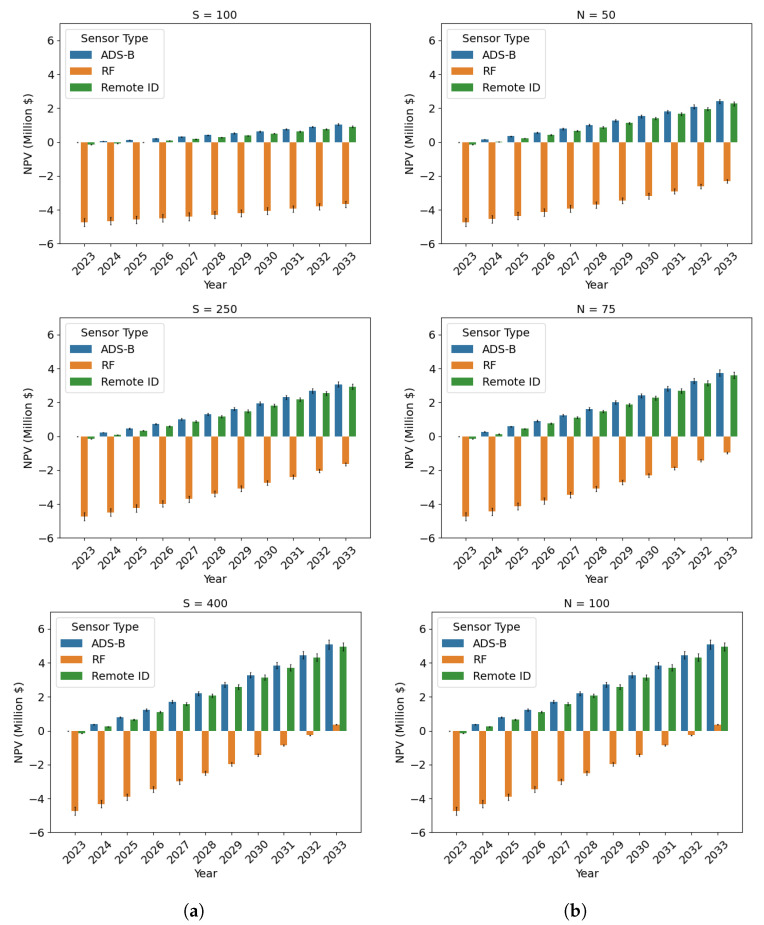
Yearly NPV for six sensor types varying *S* and *N*. (**a**) Yearly NPV for six sensor types varying *S*. (**b**) Yearly NPV for six sensor types varying *N*.

To address the uncertainty associated with the demand for AAM, it is crucial to consider a range of subscription fees and yearly numbers of subscribers instead of fixed values, as these variables directly affect the revenue and NPV generated by LASIC. Therefore, a sensitivity analysis was conducted to examine the impact of the changes in these variables on the NPV over the analysis period. For this analysis, the sensor types with the three highest-producing NPVs in the homogeneous sensor network—ADS–B, RF, and remote ID—are considered. To vary the yearly number of subscribers for LASIC, the number of subscribers in the initial year (2024) is varied, which affects the number of subscribers in the subsequent years. Based on the survey responses, three values for the monthly subscription fee per subscriber (*S*)—USD 100, USD 250, and USD 400—and three values for the number of potential subscribers in 2024 (*N*)—50, 75, and 100—are considered. The trends observed in Figure 15 show that higher values of *S* and *N* lead to increases in the NPV and cause the BEP to occur earlier. These effects are attributed to the increase in revenue generation prompted by increases in *S* and *N*. As demonstrated by the example of the RF sensor type, when *S* is USD 100, the NPV in 2033 is USD −3.67 million and cannot reach the BEP within the analysis period. On the other hand, when *S* increases to USD 400, for the same sensor type, the NPV reaches a BEP of USD 0.37 million by 2033. Similarly, if *N* is 50, the NPV shows a net loss of USD 2.31 million in 2033, and it is not possible to reach BEP during the analysis period. However, for the same sensor type, when *N* increases to 100, the NPV is expected to reach BEP of USD 3.74 million by 2033. The analysis highlights that the state government can achieve a net profit for several sensor types within the analysis period as long as *S* and *N* are not too low. The values of *S* and *N*, at which a positive NPV is ensured, are contingent on the chosen sensor type. If the objective is to detect and track solely cooperative aircraft, then ADS–B and remote ID are profitable options for the sensor types for the AAM surveillance network and LASIC, as discussed in Section 4.3. For this case, even if *S* and *N* assume values of USD 100 and 50, respectively, the state government can still attain a net profit within the analysis period. On the other hand, if both cooperative and non-cooperative aircraft are required to be tracked, then RF represents the most profitable sensor type for the AAM surveillance network and LASIC, as discussed in Section 4.3. In this scenario, the state government should ensure *S* is no less than USD 400 when *N* is 100 to achieve a net profit within the analysis period.

To understand the impact of the changes in the terrain-based sensor detection probabilities on the number of required sensors and the total sensor cost, it is important to consider that sensor detection probabilities can vary depending on the type and manufacturer of the sensor. For instance, if we focus on the radar sensor type and consider the city of Akron, we can analyze the effect of varying the sensor detection probabilities for different terrain types by conducting a sensitivity analysis. The sensor detection probabilities for different terrain types in Akron and the corresponding number of sensors and sensor cost are presented in Table 10, along with the effects of a 5% increase and a 5% decrease in sensor detection probabilities. The preset value of the sensor detection probabilities ranges from 0.75 to 0.95 for different terrain types (refer to Table 5). A 5% increase in sensor detection probabilities results in a decrease in both the number of sensors required and the total sensor cost. This is because a higher sensor detection probability allows the radar to cover the same area with fewer sensors while increasing the probability of tracking AAM aircraft, thereby reducing the sensor cost for a given sensor type. Conversely, a 5% decrease in sensor detection probabilities results in an increase in both the number of sensors required and the total sensor cost, as a lower sensor detection probability requires more sensors to cover the same area.

The sensitivity analysis of the minimum required detection probability is important because it helps to understand how the number of sensors and their cost vary with changes in *r*. A higher *r* value means that the sensor needs to be more sensitive to detect AAM aircraft with a higher level of confidence, which can increase the number of sensors required and the cost of the sensors. For example, in Table 11, provided for Akron City, we can see how the number of sensors and their cost vary for different sensor types as *r* increases. The number of sensors and the cost of a sensor type are more affected when the sensor type has a lower range, lower detection probability on all terrain types, lower field of view (higher δ), and a higher unit price. For example, the acoustic sensor type is the most affected by changes in *r*. As the value of *r* increases from 0.96 to 0.99, the cost of the acoustic sensor type increases significantly from USD 108.612 million to USD 154.350 million, and the number of sensors required also increases from 12,068 to 17,150. On the other hand, the ADS–B sensor type is the least affected by changes in *r*. We can see that for ADS–B sensors, increasing the required detection probability from 0.96 to 0.99 leads to an increase in the cost of the sensors from USD 0.002 million to USD 0.005 million and the number of sensors required from 1 to 2.

## 5. Conclusions and Future Work

To enable the real-time detection and tracking of AAM aircraft flying at lower altitudes, an effective AAM surveillance network is required to ensure adequate coverage and monitoring. Our study addresses the novel challenge of optimizing the placement of surveillance sensors for AAM. We propose the SAND model, which aims to design an AAM surveillance network that provides full coverage in a specified operational area while minimizing the total sensor cost. The model considers various factors such as sensor types, terrain types, terrain-based sensor detection probabilities, and minimum detection probability requirements. We consider two types of surveillance sensor networks: the homogeneous sensor network and the heterogeneous sensor network. Additionally, we present LASIC as a centralized cloud database that needs to be connected to the AAM surveillance network to efficiently store and process the large amounts of data generated by the network. The required features and functionalities of LASIC are determined based on AAM market data and survey inputs. To justify the investment in the AAM surveillance network and LASIC, a rigorous data-driven cost–benefit analysis was conducted by identifying, quantifying, and evaluating the costs and benefits associated with the infrastructure. We conducted the analysis for the State of Ohio over a 10-year period by estimating the NPVs for different sensor types.

The cost–benefit analysis identifies two significant cost factors—the surveillance sensor cost and the cloud-computing cost—along with a benefit factor, which is the revenue generated by LASIC. This revenue is influenced by the subscription fee and the number of potential subscribers. The cloud-computing cost for LASIC depends on the cloud server pricing policies and the required components for real-time and offline features. Due to larger areas and higher air traffic demand forecasts in Cleveland, Columbus, and Cincinnati compared to Akron, Toledo, and Dayton, the cloud-computing costs are higher in the former set of cities. According to the homogeneous sensor placement analysis, the most profitable sensor types for detecting cooperative aircraft are ADS–B and remote ID sensor types, whereas for tracking both cooperative and non-cooperative aircraft, the most profitable option is the RF sensor type. This is because these sensors have a larger range, higher field of view, higher detection probability, lower unit price, and lower cost, leading to higher NPVs compared to other sensor types. The findings also indicate that by selecting an optimal combination of sensors of different sensor types to effectively cover a given area, a lower cost and higher NPV can be achieved through heterogeneous sensor placement. Furthermore, the results show that the total sensor cost in each city of SMCO varies based on factors such as city area, shape, terrain type, and terrain-based sensor detection probabilities. Given these factors, Columbus was found to require the largest sensor cost, while Dayton had the lowest sensor cost.

Because of the uncertainties in AAM demand and the significant influence of certain parameters on the results, such as the LASIC subscription fee, number of initial subscribers, terrain-based sensor detection probabilities, and minimum required detection probability, we performed a sensitivity analysis. This analysis aimed to observe how changes in these parameters impact the results. The analysis indicates that an increase in terrain-based sensor detection probabilities leads to a decrease in the required number of sensors and total sensor cost, while a decrease in detection probability has the opposite effect. The analysis also reveals that an increase in the minimum required detection probability leads to an increase in both the number of sensors required and the total sensor cost, with the acoustic sensor type being the most affected and the ADS–B sensor type being the least affected. Furthermore, the analysis shows that higher values of subscription fees and numbers of subscribers lead to increases in the NPV generated by LASIC and cause the BEP with respect to the NPV to occur earlier, as they increase revenue generation.

This study has produced several insights related to the AAM surveillance network and LASIC and opportunities for future research that we plan to explore further. In future work, it is crucial to consider relaxing the assumptions made during this study, as doing so would render the solutions generated by the SAND model more practical. For instance, reevaluating the consideration of potential sensor obstructions will help ensure that detection requirements are met, even in the presence of natural or human-made structures in the AAM surveillance area. Additionally, it is essential to account for trajectory planning, as the trajectories of AAM flights determine the density of flights in the airspace. Sensors have a certain capacity of aircraft that they can detect at any given time within their range. The sensor placement solution should factor this in to avoid exceeding their capacities. Within this trajectory planning, the SAND model can strategically position sensors based on the expected distances between the trajectories and sensor locations, taking into account that sensor detection performance depends on the distance between the sensor and the aircraft. Furthermore, other factors that affect sensor detection performance, such as weather conditions and sensor failures, can be considered in future work. Moreover, this model can be applied to designing surveillance networks for conventional air traffic as well. Next, the location of the sensors along and across the AAM corridors connecting the major cities of a state can be identified using the model. Furthermore, the model can be modified to connect states with the potential for AAM demand, facilitating the development of the surveillance network for AAM across the US.

## Figures and Tables

**Table 2:** Parameters, indices, and decision variables in the SAND model.

**Parameters**	**Definition**
*M*	A rectangular mesh.
*F*	Transformation function of geographic coordinate system (GCS) coordinates to projected coordinate system (PCS) coordinates.
$\lambda_{p}$	Longitude of the *p*-th point in GCS.
$\phi_{p}$	Latitude of the *p*-th point in GCS.
$n_{a}$	Number of points along the x-axis of *M*.
$n_{b}$	Number of points along the y-axis of *M*.
$L_{a}$	Length of the area along the horizontal axis.
$L_{b}$	Length of the area along the vertical axis.
*L*	Block side length.
ρ	Range of a sensor.
$P^{M}$	Set of all points in *M*.
$Z^{M}$	Set of all blocks in *M*.
*T*	Set of terrain types associated with each block in $Z^{M}$.
*S*	Set of potential sensor types.
$\omega_{T}^{s}$	Detection probabilities for all combinations of terrain types in *T* and sensor types in *S*.
$\omega_{z}^{s}$	Probability of detecting an AAM aircraft with a sensor of type *s* on block *z*.
$T_{z}$	Terrain type of the *z*-th block in *T*.
I(z)	Indicator function that equals 1 if block *z* belongs to the area, and 0 otherwise.
*Q*	Number of blocks removed from $Z^{M}$.
*C*	Set of center points of blocks in *Z*.
$R_{s}$	Sensor range for a sensor of type *s* in *S*.
$d_{e,i}$	Euclidean distance between a sensor location *e* in *C* and a point *i* in $P^{M}$.
$A_{e}^{s}$	Set of coordinates of the points covered by a sensor of type *s* at location *e*.
$B_{e}^{s}$	Set of blocks covered by a sensor of type *s* at location *e*.
$\zeta_{e}^{s}$	Mean of all the probability of sensor detection values for blocks in $B_{e}^{s}$ for a sensor of type *s*.
$\tau_{e}^{s}$	Probability of misdetection of a sensor of type *s* at location *e*.
*r*	Minimum required detection probability.
$\kappa_{e}^{s}$	Number of independent sensors of type *s* required to achieve a minimum required detection probability at location *e*.
$\tau_{el}^{s}$	Probability of misdetection of sensor *l* among $\kappa_{e}^{s}$ sensors of type *s* at location *e*.
$\psi^{s}$	Cost of a sensor of type *s*.
$\delta^{s}$	Number of sensors needed for sensor type *s* to provide $360^{\circ }$ coverage at a location.
**Indices**	
*p*	*p*-th point in GCS.
*j*	*j*-th row of blocks in *M*.
*k*	*k*-th column of blocks in *M*.
*z*	*z*-th block in $Z^{M}$ and *Z*.
*e*	*e*-th candidate sensor location in *C*.
*i*	*i*-th point in $P^{M}$.
*s*	*s*-th sensor type in S.
*l*	*l*-th sensor among $\kappa_{e}^{s}$ sensors.
**Decision Variables**	
$\lambda_{e}^{s}$	Binary variable representing whether a sensor of type *s* is placed at location *e*.
$\gamma_{z}$	Binary variable representing whether block *z* is covered by at least one sensor.

**Table 3:** Types and sizes of surveillance data.

Aircraft Type	Interface Standard	Number of Data Items	Message Size (Bits)
Cooperative manned aircraft	ASTERIX CAT-021	42	1136
Cooperative uncrewed aircraft	ASTERIX CAT-129	14	432
Non-cooperative aircraft	ASTERIX CAT-062	27	2648

**Table 4:** Selected sensors from each sensor type.

Sensor Types	Vendor	System	Range (km)	ψ (≈USD)	δ
Radar	Echodyne, Kirkland, WA, USA	Echo Guard	2.41	35,000	3
ADS–B	AVIONIX Software S.L., Bigfork, MT, USA	CamelCase pingStation3	321.87	2250	1
Remote ID	BlueMark Innovations BV, Enschede, The Netherlands	Drone Scout	5.02	1100	1
RF	Dedrone, San Francisco, CA	RF-360	4.99	35,000	1
Acoustic	OptiNav, Bellevue, WA, USA	Drone Hound	0.5	9000	1
Optical Camera	Axis Communications, Lund, Sweden	Q6225-LE PTZ Network Camera	0.4	3500	6

**Table 5:** Detection probability of sensors based on different terrain types: $\omega_{T}^{s}$ matrix.

Sensor Type (*S*)	Terrain Type (*T*)				
	**Open**	**Water**	**Neighborhood**	**Hill**	**Commercial Area**
Radar	0.95	0.90	0.85	0.75	0.75
ADS–B	0.99	0.99	0.90	0.85	0.80
Remote ID	0.95	0.95	0.85	0.80	0.75
Radio Frequency	0.95	0.95	0.85	0.80	0.75
Acoustic	0.75	0.65	0.40	0.25	0.20
Optical Camera	0.90	0.90	0.80	0.75	0.70

**Table 6:** Number of sensors required in SMCO.

City	Radar	ADS–B	RF	Remote ID	Acoustic	Optical Camera
Columbus	610	1	49	49	29,500	55,000
Cleveland	261	1	24	24	10,684	21,642
Cincinnati	228	1	20	20	20,000	34,335
Toledo	192	1	17	17	15,305	33,594
Akron	153	1	14	14	13,920	27,228
Dayton	110	1	11	11	10,095	22,890

**Table 7:** Comparison of total number of sensors needed between homogeneous and heterogeneous sensor networks.

	Total Number of Sensors Needed			
	Homogeneous Sensor Network			Heterogeneous Sensor Network
City	Radar	Acoustic	Optical Camera	Radar, Acoustic, and Optical Camera
Columbus	610	29,500	55,000	648
Cleveland	261	10,684	21,642	333
Cincinnati	228	20,000	34,335	240
Toledo	192	15,305	33,594	192
Akron	153	13,920	27,228	155
Dayton	110	10,095	22,890	112

**Table 8:** Comparison of total sensor cost between homogeneous and heterogeneous sensor networks.

	Total Sensor Cost (Million USD)			
	Homogeneous Sensor Network			Heterogeneous Sensor Network
City	Radar	Acoustic	Optical Camera	Radar, Acoustic, and Optical Camera
Columbus	21.35	265.50	192.50	20.75
Cleveland	9.14	96.16	75.75	8.51
Cincinnati	7.98	180.00	120.17	7.83
Toledo	6.72	137.75	117.58	6.72
Akron	5.36	125.28	95.30	5.06
Dayton	3.85	90.86	80.12	3.52

**Table 9:** Runtime of algorithm for homogeneous and heterogeneous sensor networks.

	Runtime (Second)							
		Homogeneous Sensor Network						Heterogeneous Sensor Network
City	Number of Blocks	Radar	ADS–B	Remote ID	RF	Acoustic	Optical Camera	Radar, Acoustic, and Optical Camera
Columbus	130 × 126	68.97	198.99	48.23	51.95	45.25	38.80	230.34
Cleveland	77 × 96	14.78	29.25	8.70	10.56	5.43	6.30	56.67
Cincinnati	58 × 77	3.67	6.67	1.31	1.80	1.05	1.02	8.75
Toledo	57 × 72	2.01	5.23	1.25	1.66	0.87	0.90	7.78
Akron	65 × 62	1.45	3.11	0.87	0.89	0.03	0.03	2.03
Dayton	54 × 60	0.96	2.35	0.14	0.15	0.01	0.01	1.56

**Table 10:** Detection probability of a radar for different terrain types in Akron and the corresponding number of sensors and sensor cost, along with the effects of a 5% increase and a 5% decrease in detection probability.

Case	Terrain Type (*T*)					Number of Sensors	Total Sensor Cost (USD Million)
	**Open**	**Water**	**Neighborhood**	**Hill**	**Commercial Area**		
Preset Value	0.9500	0.9000	0.8500	0.7500	0.7500	153	5.355
5% Increase	0.9975	0.945	0.8925	0.7875	0.7875	114	3.990
5% Decrease	0.9025	0.855	0.8075	0.7125	0.7125	165	5.775

**Table 11:** Number of sensors and cost for different sensor types at different *r* values.

Sensor Type	*r*	Number of Sensors	Total Sensor Cost (Million USD)
ADS–B	0.96	1	0.002
	0.97	1	0.002
	0.98	1	0.002
	0.99	2	0.005
Remote ID	0.96	10	0.011
	0.97	11	0.012
	0.98	14	0.015
	0.99	15	0.017
Radar	0.96	117	4.095
	0.97	120	4.200
	0.98	153	5.355
	0.99	165	5.775
RF	0.96	10	0.350
	0.97	11	0.385
	0.98	14	0.490
	0.99	15	0.525
Acoustic	0.96	12,068	108.612
	0.97	12,421	111.789
	0.98	13,920	125.280
	0.99	17,150	154.350
Optical Camera	0.96	19,986	69.951
	0.97	26,742	93.597
	0.98	27,228	95.298
	0.99	28,680	100.380

## Data Availability

Data is contained within the article.
